# Supplementary material for: Predictors of specialist somatic healthcare utilization among older people with intellectual disability and their age-peers in the general population: a national register study
Source: BMJ Open. 2023 Jul 4;13(7):e072679. doi: 10.1136/bmjopen-2023-072679 (PMC10335550; doi:10.1136/bmjopen-2023-072679)
Supplement: Author's manuscript [file bmjopen-2023-072679.draft_revisions.pdf]

BMJ Open

BMJ Open is committed to open peer review. As part of this commitment we make the peer review history of every article we publish publicly available.

When an article is published we post the peer reviewers' comments and the authors' responses online. We also post the versions of the paper that were used during peer review. These are the versions that the peer review comments apply to.

The versions of the paper that follow are the versions that were submitted during the peer review process. They are not the versions of record or the final published versions. They should not be cited or distributed as the published version of this manuscript.

BMJ Open is an open access journal and the full, final, typeset and author-corrected version of record of the manuscript is available on our site with no access controls, subscription charges or pay-per-view fees (http://bmjopen.bmj.com).

If you have any questions on BMJ Open's open peer review process please email info.bmjopen@bmj.com

BMJ Open

Predictors of specialist somatic healthcare utilization among older people with intellectual disability and their age-peers in the general population: A national register study

Journal:	*BMJ Open*
Manuscript ID	bmjopen-2023-072679
Article Type:	Original research
Date Submitted by the Author:	13-Feb-2023

Complete List of Authors:	Sandberg, Magnus; Lunds Universitet, Department of Health Sciences<br>Axmon, Anna; Lunds University, EPI@LUND (Epidemiology, Population studies and Infrastructures at Lund University)<br>Ahlström, Gerd; Lunds Universitet, Health Sciences<br>Kristensson, Jimmie; Lunds Universitet, Department of Health Sciences; Lund University, Institute for Palliative Care

Keywords:	Organisation of health services < HEALTH SERVICES ADMINISTRATION & MANAGEMENT, Aged, REGISTRIES, Health Services Accessibility

SCHOLARONE™ Manuscripts

I, the Submitting Author has the right to grant and does grant on behalf of all authors of the Work (as defined in the below author licence), an exclusive licence and/or a non-exclusive licence for contributions from authors who are: i) UK Crown employees; ii) where BMJ has agreed a CC-BY licence shall apply, and/or iii) in accordance with the terms applicable for US Federal Government officers or employees acting as part of their official duties; on a worldwide, perpetual, irrevocable, royalty-free basis to BMJ Publishing Group Ltd ("BMJ") its licensees and where the relevant Journal is co-owned by BMJ to the co-owners of the Journal, to publish the Work in this journal and any other BMJ products and to exploit all rights, as set out in our licence.

The Submitting Author accepts and understands that any supply made under these terms is made by BMJ to the Submitting Author unless you are acting as an employee on behalf of your employer or a postgraduate student of an affiliated institution which is paying any applicable article publishing charge ("APC") for Open Access articles. Where the Submitting Author wishes to make the Work available on an Open Access basis (and intends to pay the relevant APC), the terms of reuse of such Open Access shall be governed by a Creative Commons licence – details of these licences and which Creative Commons licence will apply to this Work are set out in our licence referred to above.

Other than as permitted in any relevant BMJ Author's Self Archiving Policies, I confirm this Work has not been accepted for publication elsewhere, is not being considered for publication elsewhere and does not duplicate material already published. I confirm all authors consent to publication of this Work and authorise the granting of this licence.

For peer review only

Predictors of specialist somatic healthcare utilization among older people with intellectual disability and their age-peers in the general population: A national register study

Magnus Sandberg [a*], Anna Axmon [b], Gerd Ahlström [a], Jimmie Kristensson [a,c]

[a] Department of Health Sciences, Faculty of Medicine, Lund University, P.O. Box 157, Lund SE-221 00, Sweden

[b] EPI@LUND (Epidemiology, Population studies and Infrastructures at Lund University), Division of Occupational and Environmental Medicine, Lund University, Biskopsgatan 9, Lund SE-223 62, Sweden

c. Institute for Palliative Care, Lund University, Region Skåne

* Corresponding author:

Magnus Sandberg, Department of Health Sciences, Faculty of Medicine, Lund University, P.O. Box 157, SE-221 00 Lund, Sweden E-mail: Magnus.Sandberg@med.lu.se. Tel.: +46 46 222 19 71

For peer review only

Abstract

Objectives: To compare somatic healthcare utilization among older people with ID to that of their age-peers in the general population, taking into account health and demographic factors, and to identify predictors for somatic healthcare utilization among older people with ID.

Participants: Equally sized cohorts, one with people with ID and one referent cohort, one-to-one-matched by sex and year of birth, were created. Each cohort comprised 7936 people aged 55+ years at the end of 2012.

Design: Retrospective register-based study.

Setting: All specialist in- and outpatient healthcare clinics in Sweden.

Outcome measures: Data regarding planned/unplanned and inpatient/outpatient specialist healthcare were collected from the Swedish National Patient Register for 2002-2012. Diagnoses, previous healthcare utilization, sex, age, and cohort affiliation was used to investigate potential impact on healthcare utilization.

Results: Compared to the referent cohort, the ID cohort were more likely to have unplanned inpatient and outpatient care but less likely to have planned outpatient care. Within the ID cohort, sex, age and previous use of healthcare predicted healthcare utilization.

Conclusions: Older people with ID seem to have lower risks of planned outpatient care compared to the general population that could not be explained by diagnoses. Potential explanations are that people with ID suffer from communication difficulties and experience the healthcare environment as unfriendly. Moreover, that healthcare staff lack knowledge about the particular needs of people with ID. Altogether, this may lead to people with ID being exposed to discrimination. Although these problems are known, few interventions have been evaluated, especially related to planned outpatient care.

For peer review only - http://bmjopen.bmj.com/site/about/guidelines.xhtml

Keywords: healthcare utilisation, health services research, intellectual disability, older adults, Register studies

Strengths and limitations of this study

- A strength is that as far as we know, this is the first study to investigate predictors of somatic healthcare utilization among people with ID compared to the general population.
- Another strength is that the study uses a national registers with high validity data.
- A limitation of the study is that primary healthcare data are not included.
- Another limitation is that both the ID and the referent cohort consist of healthy survivors.

Word count

3247 (excluding the title page, abstract, tables, acknowledgements, authors' contributions, and references)

For peer review only

Background

A range of chronic diseases are found to a higher degree among people with intellectual disability (ID) than in the general population. These include e.g. diabetes [1,2], cardiovascular diseases [3], osteoporosis [4], visual and hearing impairment [5,6], musculoskeletal diseases [6], and thyroid conditions [5].

Recent decades have seen an increasing life span among people with ID, resulting in an increasing number of older people with ID [7]. A consequence of this is that people with ID live longer with chronic diseases, and it is well known that older people with ID experience high rates of multi-morbidity [8,9]. Thus, older people with ID would be expected to have greater healthcare needs and utilization than their age-peers in the general population. Even so, they have been found to have lower rates of specialist healthcare, especially in higher ages [10]. A possible explanation for this is that healthcare is not necessarily sought at the right care level, i.e., whereas older people in the general population are often referred to specialist care, older people with ID are treated in primary care. Another reason may be that some disorders are underdiagnosed/treated among older people with ID. For example, many pain and psychiatric diagnoses depend on the ability of the patient to communicate symptoms, which may be difficult for people of more severe ID [11,12].

Previous studies have reported that older people in the general population with physical as well as social unmet needs are often admitted to acute medical wards [13,14]. Thus, having a high amount of unplanned care and low of planned care could be an indication of unmet needs. To enable provision of proper healthcare to older people with ID, it is important to investigate factors that may predict different forms of healthcare utilization.

Previous studies regarding somatic healthcare utilization among people with ID have demonstrated associations between a range of demographic (e.g. age, disability level), socioeconomic (e.g. income, living arrangements), health (e.g. subjective and self-perceived

For peer review only

For peer review only - http://bmjopen.bmj.com/site/about/guidelines.xhtml

health status), and treatment (e.g. polypharmacy) factors on one hand, and hospital admissions [15-19] and outpatient care [20-22] on the other. There are also studies investigating various healthcare utilization patterns [23 24]. However, the majority of these studies are cross-sectional without the possibility to investigate predictors of healthcare utilization. In addition, they investigate only subgroups of the ID population, for example those at day care centres [16], admitted for orthopaedic procedures [23] or enrolled in a home and community-based waiver [20]. In one study [17] that included both in- and outpatient data outpatient, visits was only measured with a dichotomised variable, more than 24 visits, or 24 or less visits. One exception is a comprehensive English study [24] investigating emergency department admissions and annual health check among 21 859 adults with ID compared with 152 846 age-, gender- and practice-matched controls without ID in 2009–2013. They found that people with ID were more likely than the general population to have emergency department admissions, and for reasons that were more likely to be deemed as preventable also after adjusting for nine different comorbidities. They also found that annual health checks for adults with ID did not have any effects on emergency department admissions compared to controls [24]. But also here, they only investigated certain healthcare types and diagnoses. Hence, there is a need for studies investigating somatic healthcare utilization over a longer period.

The aims of the present study were to 1) compare somatic specialist healthcare utilization among older people with ID to that of their age-peers in the general population, taking into account a range of health and demographic variables and 2) develop a model for prediction of healthcare utilization among older people with ID.

For peer review only - http://bmjopen.bmj.com/site/about/guidelines.xhtml

Material and Methods

Study design

This was a longitudinal, retrospective, population register-based study. The study was approved by the Regional Ethical Review Board in Lund (diary no 2013/15), and performed in accordance with the Helsinki declaration [25].

Setting

Sweden has a welfare system mainly funded by taxes [26]. People with permanent functional impairments can receive support from the municipality according to the Act concerning Support and Service for Persons with Certain Functional Impairments (LSS) [27]. The support available is regulated in the LSS act, and includes e.g., daytime activities and residential care. It also includes support to next of kin, for example relief services. All provided support is recorded in the national LSS-register, which is maintained by the Swedish National Board of Health and Welfare. Reporting to this register is mandatory for all municipalities. The LSS act distinguishes between three types of functional impairments, whereof one is those that have been present since birth or early years. These comprise ID and autism spectrum disorders (ASD). Thus, it is possible to separate this group of people from those with intellectual functional impairment acquired in adult life, for example caused by traumatic brain injury.

Specialist healthcare in outpatient and inpatient facilities, primary care, rehabilitation and advanced home care provided by county councils is regulated by the Health and Medical Services Act (HSL) [28]. Specialist inpatient and outpatient healthcare provided according to the HSL is registered in the National Patient Register (NPR). This register contains information on inpatient episodes and outpatient visits, with one primary and up to 21 secondary

For peer review only

diagnoses recorded according to ICD-10 (International Statistical Classification of Diseases and Related Health Problems 10th Revision) [29] at discharge. Data are also available on whether the healthcare episode was planned (i.e., if an appointment was made beforehand) or unplanned. The dates of hospital admission and discharge are recorded for inpatient healthcare, and the date of the visit for outpatient specialist healthcare. In this study, only data from somatic healthcare were used, but nonetheless, these could include both somatic and psychiatric diagnoses.

Study populations

The ID cohort comprised all people aged 55 years and older who received support and social services according to the LSS act [27] during 2012, were recorded as belonging to the group of people with functional impairment since birth or early age (i.e. having ID and/or ASD), and alive at the end of 2012. The cut-off at 55 years was chosen to define “old” because the aging process can start earlier in people with ID [30]. A referent cohort from the general population (gPop cohort) was established through one-to-one matching by birth year and sex from the Swedish National Population Register [31]. The matching was performed by Statistics Sweden [32]. In total, 15 872 people were included in the study, 7936 in the ID cohort and an equal number in the gPop cohort. If a person was included in the ID cohort, they could not be matched as a referent.

Material

Pseudonymized in- and outpatient healthcare data for all individuals in the two cohorts were collected from the NPR for the 11-year period from January 1, 2002, to December 31, 2012. An individual could have several registrations for one inpatient period because of a

For peer review only - http://bmjopen.bmj.com/site/about/guidelines.xhtml

change in ward during the hospital stay. In this paper, data on all registrations and their corresponding diagnoses are used.

Patient and public involvement

Patients and/or the public were not involved in the design, or conduct, or reporting or dissemination plans of this research. However, representatives from a patient organisation FUB, The Swedish National Association for People with Intellectual Disability, which is an advocacy organization working to enable people with an ID to live a good life, has been included in the project's reference group.

Statistical analysis

Potential impact of diagnoses, previous healthcare utilization, sex, age, and cohort affiliation (aim 1 only) on the four types of healthcare utilization (planned/unplanned, inpatient/outpatient healthcare) was evaluated using generalized linear models (GLMs) with Poisson distribution and log link, thus estimating relative risks (RRs) with 95% confidence intervals (CI). Calendar year was used to indicate repeated measures. A similar method was employed to evaluate aim 2, with the exception that only data from the ID cohort were used and thus cohort affiliation was not included in any of the models.

Diagnoses were investigated on block level, as defined in ICD-10. Yearly inpatient healthcare utilization was categorized as 0, 1, 2, or 3+ visits, whereas yearly outpatient healthcare utilization was categorized as 0, 1, 2-5, 6-10, or 11+ visits. Age was aggregated to 10-year intervals.

Both aims and all four types of healthcare utilization were investigated using similar procedures (Figure 1): First, bivariate analyses were performed to investigate possible associations between different diagnoses and healthcare utilization. Second, for each type of

For peer review only - http://bmjopen.bmj.com/site/about/guidelines.xhtml

healthcare and aim, a multivariate model was employed, including all diagnoses with $p<0.10$ in the bivariate models. Third, all diagnoses with $p<0.10$ in the multivariate model were included in final models (one per type of healthcare and aim) which also included cohort affiliation (aim 1 only), sex, age, and healthcare utilization the previous year. Fourth, the predictive ability of two different models was assessed using the Area Under the Curve (AUC).

In the final model for planned inpatient care in the ID cohort, the Hessian matrix was singular and the convergence criteria were not satisfied. We therefore ran collinearity diagnostics. This was done in a plain regression model as the GML module in SPSS does not provide this option. Diagnostic chapters were removed from the final model one by one according to their VIF-value until the convergence criteria were satisfied. Hence, these blocks were removed due to multicollinearity: A50-A64 (Infections with a predominantly sexual mode of transmission), P10-P15 (Birth trauma), Q60-Q64 (Congenital malformations of the urinary system), B50-B64 (Protozoal diseases), G50-G59 (Nerve, nerve root and plexus disorders), K70-K77 (Diseases of liver), D00-D09 (In situ neoplasms), K65-K67 (Diseases of peritoneum), G20-G26 (Extrapyramidal and movement disorders), J30-J39 (Other diseases of upper respiratory tract), I80-I89 (Diseases of veins, lymphatic vessels and lymph nodes, not elsewhere classified), K50-K52 (Noninfective enteritis and colitis), J95-J99 (Other diseases of the respiratory system), M80-M94 (Osteopathies and chondropathies), R30-R39 (Symptoms and signs involving the urinary system), K80-K87 (Disorders of gallbladder, biliary tract and pancreas), K40-K46 (Hernia), G80-G83 (Cerebral palsy and other paralytic syndromes), F70-F79 (Mental retardation), M60-M79 (Soft tissue disorders), I20-I25 (Ischemic heart diseases), M00-M25 (Arthropathies), and Z40-Z54 (Persons encountering health services for specific procedures and healthcare).

All analyses were made in IBM SPSS statistics Version 23.

For peer review only

For peer review only - http://bmjopen.bmj.com/site/about/guidelines.xhtml

<<Insert Figure 1 about here>>

Results

In multivariate models including both cohorts, people in the ID cohort had increased risk of unplanned care – both inpatient and outpatient – compared to the gPop cohort (RR 1.40; 95% CI 1.33-1.48, and RR 1.14; 95% CI 1.08-1.20, respectively). However, those with ID had decreased risk of planned outpatient care (RR 0.91; 95% CI 0.89-0.94). There was no change in risk for planned inpatient care (RR 1.01; 95% CI 0.94-1.09).

Within the ID cohort, men had decreased risk of unplanned outpatient care as well as planned care (both inpatient and outpatient) compared to women (RR 0.93, 95% CI 0.88-0.99, RR 0.92; 95% CI 0.85-0.99, and RR 0.92; 95% CI 0.88-0.95 respectively; Table 1). Increasing age was associated with increased risk of inpatient care, both planned and unplanned, but with decreased risk of planned outpatient care (Table 1). Again, with only a few exceptions, healthcare the previous year carried an increased risk of healthcare utilization (Table 1). No diagnosis increased the risk of any type of healthcare (Table 2).

All models, i.e. based on ID cohort only, and for all four types of care, were able to predict healthcare utilization better than chance (Figure 2).

<<Insert Table 1 about here>>

<<Insert Table 2 about here>>

<<Insert Figure 2 about here>>

For peer review only - http://bmjopen.bmj.com/site/about/guidelines.xhtml

Discussion

Even when adjusting for demographic factors, all recorded diagnoses, and previous healthcare utilization, ID was associated with increased unplanned healthcare utilization. If unplanned healthcare is a measure of unmet healthcare needs, this is cause for concern. Another important finding was the lower healthcare utilization among men compared to women among people with ID, even when all recorded diagnoses were taken into account. This suggests gender differences in access to healthcare among older people with ID.

The major strength of the present study is the use of national registers with high validity to identify people with ID as well as healthcare utilization and diagnoses. In using data from 11 consecutive years and repeated measures analyses, we were able to investigate healthcare utilization following specific diagnoses, rather than mere associations (i.e. non-directional), as well as adjust for previous healthcare utilization. Thus, the results presented for demographic factors and diagnoses are most likely not inflated by people with particularly high healthcare consumption. In addition, reporting of inpatient- and specialist outpatient care to NPR is mandatory for healthcare providers and the register has been shown to have high validity [33].

A potential weakness with the present study is the use of an administrative ID cohort. The LSS register comprises all people who have received service and support aimed at people with ID and/or ASD, including support to informal caregivers. However, those who did not receive such support in 2012 were not included in the ID cohort. This may include, for example, people with ID who are being taken care of solely by parents or other family without support from the municipality. Even so, the Swedish welfare system is designed so that vulnerable groups are cared for by the state, county, or municipality. It is rare that informal caregivers within the family do not receive any support at all. In addition, considering the age group studied, we believe that the number of people with ID being cared for by their parents is low. As the data were collected retrospectively for people alive at the

For peer review only - http://bmjopen.bmj.com/site/about/guidelines.xhtml

end of the study period, another potential weakness could be cohort effects, i.e., that people with ID that survived and turned 55 years or older might be healthier than their younger non-surviving peers.

We collected information on healthcare utilization as well as diagnoses from the NPR. Although this register has national coverage of specialist care, it does not include visits to primary care. Thus, even though we have good coverage of specialist healthcare utilization, it is difficult to interpret the results in terms of unmet *overall* healthcare needs. As people with ID are less likely than those in the general population to get referrals from primary to specialist care [34] any bias introduced from the lack of primary healthcare data should have caused an underestimation of the higher healthcare utilization associated with having ID. In addition, the diagnoses are aggregated over an 11-year period and no consideration was taken to multi-morbidity. To get a deeper understanding of the determinants of unplanned healthcare and the adequacy of the care, a limited number of diagnoses, or combinations of diagnoses, should be investigated more in detail in future studies.

One of our main findings is that people with ID have less planned outpatient care than the general population, with lower risks in higher ages, which is not explained by differences in diagnoses. This is surprising as people with ID have a more complex disease panorama than the general population [4-6 9], and as the prevalence of multiple chronic diseases is a well-known explanation for higher amount of healthcare utilization in the general older population [35]. In addition, it is reasonable to assume that unplanned care in terms of hospital admissions are followed up with planned forms of care and services in order to prevent readmission [36]. Previous research has also found that annual health checks seem to reduce preventable emergency admissions [24]. Only a few studies have investigated healthcare use among people with ID compared to that in the general population [37-42]. Of these, only Skorpen, et al. [41] reported results consistent with the present study, with adults with ID being hospitalised more

For peer review only - http://bmjopen.bmj.com/site/about/guidelines.xhtml

frequently at younger age and less frequently at older ages compared to the general population. Most studies did not consider the influence of age on healthcare utilization [37 38 40 42]. However, Hsu, et al. [39] investigated ambulatory care visits among older people (65 years and older) with and without ID, and found that people with ID had a higher mean number of visits and mean annual visits, and that age group affected the annual visits with middle-elderly (74-84) in both groups having the highest frequency of visits. There is a range of possible explanations for the lower rates of planned healthcare among people with ID. These include difficulties accessing the healthcare system [43-45] and lower rates of planned preventive healthcare, such as breast and cervical cancer screening [46 47]. Furthermore, even when having entered the healthcare system, people with ID may have communication difficulties and experience the healthcare environment as unfriendly [48-50]. An explanation of particular concern is that the ID population is discriminated by the healthcare system [51].

In addition to being discriminated in relation to the general population, there could also be discrimination between different groups within the ID population. The present study also revealed that women with ID have higher healthcare utilization than men with ID. Gender differences in psychiatric diagnoses have been reported [52], but do not seem to have been investigated when it comes to somatic healthcare utilization. Thus, it is important to investigate whether males with ID are at risk for a 'double discrimination'.

Even though the need for interventions regarding requirement of knowledge about and various aspects of supporting people with ID have been addressed since the 1990'ies [53], there is a lack of interventions studies and the reduced access to healthcare among people with ID still persists today [48 54]. In addition, adults with ID have been reported to be at high risk for preventable unplanned admissions, despite having similar primary care utilization before admission as the general population [55]. Hence, further interventions are needed, e.g. such that focus on communication, knowledge/information and profession-specific needs [53]. This may

For peer review only - http://bmjopen.bmj.com/site/about/guidelines.xhtml

include enhancements of Medical- and Nursing Schools curriculums [56-58] to increase knowledge about people with ID, which may facilitate access to healthcare, which in turn may support the health and wellbeing among people with ID. Moreover, interventions aimed at primary care providers may be relevant, as these often are the gate keepers to other forms of care and often have continuous contact with the person with ID, and thus, have the possibility to coordinate care, and also to provide disease prevention, early detection, and appropriate management [59]. People with ID in primary care have been reported to have lower continuity of care and be less likely to have longer appointments than the general population, which could be areas of improvement [60], especially as higher continuity of care has been found to be associated to emergency department visits among people with ID [61]. Even though initiatives have been taken to increase the number of people with ID that seek primary healthcare [56 62 63], the effects of these interventions do not seem to have been evaluated. Furthermore, few studies have evaluated continuity of care interventions for people with ID and none with unplanned admissions as primary outcome [64]. Thus, this needs to be addressed in future research.

Conclusions

People with ID have less planned outpatient healthcare than the general population. This difference was not explained by diagnoses, demographic factors, or previous healthcare utilization. Thus, it is likely a result of other factors, for example communication difficulties and experiences of the healthcare environment as unfriendly among people with ID, as well as a lack of knowledge about ID among healthcare staff, and discrimination of people with ID. Discrimination could also be the reason for less planned and more unplanned inpatient and outpatient specialist care among those with ID. These problems have been addressed for a long time, but there are still few intervention studies in the area and especially studies

For peer review only - http://bmjopen.bmj.com/site/about/guidelines.xhtml

targeting the problems related to planned healthcare identified in the present study. Thus, there is a great need for evaluation of healthcare interventions, especially in planned outpatient care such as primary care, which aim to give people with ID access to healthcare to the same extent as the general population.

Acknowledgements

We would like to acknowledge FORTE – The Swedish Research Council for Health, Working Life and Welfare (grant no. 2014–4753) and the Faculty of Medicine, Lund University, Sweden for financing this study.

Ethics statements

Patient consent for publication

Not applicable.

Ethics approval

Approval was obtained from the Regional Ethical Review Board in Lund (reg no 2013/15). The Swedish National Board of Health and Welfare as well as Statistics Sweden performed a separate secrecy review in 2014 before providing access to the data. All analyses were performed using pseudonymized datasets.

Funding statement

This work was funded by Forte, the Swedish Research Council for Health, Working Life and Welfare (http://forte.se/en) no. 2014-4753 with GA as PI. The funders had no role in study design, data collection and analysis, decision to publish, or preparation of the

For peer review only

manuscript.

Data sharing statement

The data in the present study contains sensitive information on a very vulnerable group, i.e., people with ID. However, as our database is compiled by register data only, other researchers may contact Statistics Sweden and the Swedish National Board of Health and Welfare to get access to the different registers included, and thereby recreate the data

Author contributions

MS participated in the design of the study and in the collection of data, performed the analysis and interpretation of data, and drafted the manuscript. GA participated in the design of the study and helped in the interpretation of the data and drafting of the manuscript. AA participated in the design of the study and helped in the interpretation of the data and drafting of the manuscript. JK participated in the design of the study and helped in the interpretation of the data in drafting the manuscript. All authors read and approved the final manuscript.

Conflict of Interests Statements

The authors have declared that no competing interests exist.

For peer review only - http://bmjopen.bmj.com/site/about/guidelines.xhtml

References

1. Axmon A, Ahlstrom G, Hoglund P. Prevalence and treatment of diabetes mellitus and hypertension among older adults with intellectual disability in comparison with the general population. *BMC Geriatr* 2017;17(1):272. doi: 10.1186/s12877-017-0658-2 [published Online First: 2017/11/25]
2. Flygare Wallen E, Ljunggren G, Carlsson AC, et al. High prevalence of diabetes mellitus, hypertension and obesity among persons with a recorded diagnosis of intellectual disability or autism spectrum disorder. *J Intellect Disabil Res* 2018;62(4):269-80. doi: 10.1111/jir.12462 [published Online First: 2017/12/28]
3. Draheim CC. Cardiovascular disease prevalence and risk factors of persons with mental retardation. *Mental retardation and developmental disabilities research reviews* 2006;12(1):3-12.
4. van Schrojenstein Lantman-De Valk HM, Van Den Akker M, Maaskant MA, et al. Prevalence and incidence of health problems in people with intellectual disability. *J Intellect Disabil Res* 1997;41(1):42-51.
5. Kapell D, Nightingale B, Rodriguez A, et al. Prevalence of chronic medical conditions in adults with mental retardation: comparison with the general population. *Ment Retard* 1998;36(4):269-79. doi: 10.1352/0047-6765(1998)036<0269:pocmci>2.0.co;2 [published Online First: 1998/08/26]
6. van Schrojenstein Lantman-De Valk HM, Metsemakers JF, Haveman MJ, et al. Health problems in people with intellectual disability in general practice: a comparative study. *Fam Pract* 2000;17(5):405-7. [published Online First: 2000/10/06]
7. Fisher K, Kettl P. Aging with mental retardation: increasing population of older adults with MR require health interventions and prevention strategies. *Geriatrics* 2005;60(4):26-29.
8. McCarron M, Swinburne J, Burke E, et al. Patterns of multimorbidity in an older population of persons with an intellectual disability: results from the intellectual disability supplement to the Irish longitudinal study on aging (IDS-TILDA). *Res Dev Disabil* 2013;34(1):521-7. doi: 10.1016/j.ridd.2012.07.029 [published Online First: 2012/10/23]
9. Sandberg M, Ahlstrom G, Kristensson J. Patterns of Somatic Diagnoses in Older People with Intellectual Disability: A Swedish Eleven Year Case-Control Study of Inpatient Data. *Journal of applied research in intellectual disabilities : JARID* 2017;30(1):157-71. doi: 10.1111/jar.12230 [published Online First: 2015/11/07]
10. Sandberg M, Ahlstrom G, Axmon A, et al. Somatic healthcare utilisation patterns among older people with intellectual disability: an 11-year register study. *BMC Health Serv Res* 2016;16(1):642. doi: 10.1186/s12913-016-1880-x [published Online First: 2016/11/11]
11. Axmon A, Ahlström G, Westergren H. Pain and Pain Medication among Older People with Intellectual Disabilities in Comparison with the General Population. *Healthcare* 2018;6(2):67.
12. Boardman L, Bernal J, Hollins S. Communicating with people with intellectual disabilities: a guide for general psychiatrists. *Advances in psychiatric treatment* 2014;20(1):27-36.
13. Campbell SE, Seymour DG, Primrose WR. A systematic literature review of factors affecting outcome in older medical patients admitted to hospital. *Age Ageing* 2004;33(2):110-5. doi: 10.1093/ageing/afh036 [published Online First: 2004/02/13]

For peer review only - http://bmjopen.bmj.com/site/about/guidelines.xhtml

14. Molloy GJ, McGee HM, O'Neill D, et al. Loneliness and emergency and planned hospitalizations in a community sample of older adults. *J Am Geriatr Soc* 2010;58(8):1538-41. doi: 10.1111/j.1532-5415.2010.02960.x [published Online First: 2010/07/22]

15. Lin JD, Loh CH, Yen CF, et al. Medical care services for people with intellectual disabilities living in the general community: a cross-sectional survey of inpatient care utilization in Taiwan, 2001. *Disabil Rehabil* 2007;29(18):1411-6. doi: 10.1080/09638280600999576 [published Online First: 2007/08/31]

16. Lin JD, Wu JL, Lee PN. Utilization of inpatient care and its determinants among persons with intellectual disabilities in day care centres in Taiwan. *J Intellect Disabil Res* 2004;48(Pt 7):655-62. doi: 10.1111/j.1365-2788.2003.00572.x [published Online First: 2004/09/11]

17. Loh CH, Lin JD, Choi IC, et al. Longitudinal analysis of inpatient care utilization among people with intellectual disabilities: 1999-2002. *J Intellect Disabil Res* 2007;51(Pt 2):101-8. doi: 10.1111/j.1365-2788.2006.00822.x [published Online First: 2007/01/16]

18. Merrick J, Davidson PW, Morad M, et al. Older adults with intellectual disability in residential care centers in Israel: health status and service utilization. *Am J Ment Retard* 2004;109(5):413-20. doi: 10.1352/0895-8017(2004)109<413:oawidi>2.0.co;2 [published Online First: 2004/08/10]

19. Venkat A, Pastin RB, Hegde GG, et al. An analysis of ED utilization by adults with intellectual disability. *Am J Emerg Med* 2011;29(4):401-11. doi: 10.1016/j.ajem.2009.11.009 [published Online First: 2010/09/10]

20. Hall A, Wood D, Hou T, et al. Patterns in primary health care utilization among individuals with intellectual and developmental disabilities in Florida. *Intellectual and developmental disabilities* 2007;45(5):310-22. doi: 10.1352/0047-6765(2007)45[310:piphcu]2.0.co;2 [published Online First: 2007/09/25]

21. Lin JD, Wu JL, Lee PN. Healthcare needs of people with intellectual disability in institutions in Taiwan: outpatient care utilization and implications. *J Intellect Disabil Res* 2003;47(Pt 3):169-80. [published Online First: 2003/02/27]

22. Lin JD, Yen CF, Loh CH, et al. A cross-sectional study of the characteristics and determinants of emergency care utilization among people with intellectual disabilities in Taiwan. *Res Dev Disabil* 2006;27(6):657-67. doi: 10.1016/j.ridd.2005.09.001 [published Online First: 2005/11/10]

23. Bathje M, Conrad S, Medick M, et al. Differences in Hospital-Based Care for Patients With Intellectual and Developmental Disabilities. *Am J Occup Ther* 2021;75(3) doi: 10.5014/ajot.2021.046508

24. Carey IM, Hosking FJ, Harris T, et al. Health Services and Delivery Research. An evaluation of the effectiveness of annual health checks and quality of health care for adults with intellectual disability: an observational study using a primary care database. Southampton (UK): NIHR Journals Library

25. World Medical Association. World medical association declaration of helsinki: Ethical principles for medical research involving human subjects. *JAMA* 2013;310(20):2191-94. doi: 10.1001/jama.2013.281053

26. Anell A, Glenngard AH, Merkur SM. Sweden: Health system review. *Health systems in transition* 2012;14(5):1-159.

27. *Lag om stöd och service till vissa funktionshindrade (LSS) [Act concerning Support and Service for Persons with Certain Functional Impairments]*. SFS 1993:387. Stockholm: Socialdepartementet. Available: https://www.riksdagen.se/sv/dokument-

For peer review only - http://bmjopen.bmj.com/site/about/guidelines.xhtml

lagar/dokument/svensk-forfattningssamling/lag-1993387-om-stod-och-service-till-vissa_sfs-1993-387 [Accessed 08 Feb 2023].
28. *Hälso- och sjukvårdslag* [*The Health and Medical Service Act*]. SFS 2017:30. Stockholm: Socialdepartementet. Available: https://www.riksdagen.se/sv/dokument-lagar/dokument/svensk-forfattningssamling/halso--och-sjukvardslag_sfs-2017-30 [Accessed 08 Feb 2023].
29. World Health Organisation. International Statistical Classification of Diseases and Related Health Problems, 10th Revision, 2015 Version Geneva2015 [Available from: https://icd.who.int/browse10/2019/en#/ [Accessed 08 Feb 2023].
30. Thorpe L, Davidson PW, Janicki MP. Healthy Ageing – Adults with Intellectual Disabilities: Biobehavioural Issues. *Journal of Applied Research in Intellectual Disabilities* 2001;14:218-28.
31. Wilén A, Johannesson I. A new Total population register system: More possibilities and better quality. Örebro, 2002.
32. Statistics Sweden. Statistic Sweden startpage 2019 [Available from: http://www.scb.se/en_/ [Accessed 08 Feb 2023].
33. Ludvigsson JF, Andersson E, Ekbom A, et al. External review and validation of the Swedish national inpatient register. *BMC Public Health* 2011;11:450. doi: 1471-2458-11-450 [published Online First: 2011/06/11]
34. Weise J, Pollack A, Britt H, et al. Primary health care for people with an intellectual disability: an exploration of consultations, problems identified, and their management in Australia. *J Intellect Disabil Res* 2017;61(5):399-410. doi: 10.1111/jir.12352 [published Online First: 2017/01/25]
35. Lehnert T, Heider D, Leicht H, et al. Review: health care utilization and costs of elderly persons with multiple chronic conditions. *Med Care Res Rev* 2011;68(4):387-420. doi: 10.1177/1077558711399580 [published Online First: 2011/08/05]
36. Feigenbaum P, Neuwirth E, Trowbridge L, et al. Factors contributing to all-cause 30-day readmissions: a structured case series across 18 hospitals. *Med Care* 2012:599-605.
37. Balogh RS, Hunter D, Ouellette-Kuntz H. Hospital Utilization among Persons with an Intellectual Disability, Ontario, Canada, 1995–2001. *Journal of Applied Research in Intellectual Disabilities* 2005;18(2):181-90. doi: 10.1111/j.1468-3148.2005.00247.x
38. Beange H, McElduff A, Baker W. Medical disorders of adults with mental retardation: a population study. *Am J Ment Retard* 1995;99(6):595-604. [published Online First: 1995/05/01]
39. Hsu SW, Lin JD, Chiang PH, et al. Comparison of outpatient services between elderly people with intellectual disabilities and the general elderly population in Taiwan. *Res Dev Disabil* 2012;33(5):1429-36. doi: 10.1016/j.ridd.2012.03.014 [published Online First: 2012/04/24]
40. Lunsky Y, Lin E, Balogh R, et al. Are adults with developmental disabilities more likely to visit EDs? *Am J Emerg Med* 2011;29(4):463-5. doi: 10.1016/j.ajem.2010.12.028 [published Online First: 2011/02/15]
41. Skorpen S, Nicolaisen M, Langballe EM. Hospitalisation in adults with intellectual disabilities compared with the general population in Norway. *J Intellect Disabil Res* 2016 doi: 10.1111/jir.12255 [published Online First: 2016/02/26]
42. Straetmans JM, van Schrojenstein Lantman-de Valk HM, Schellevis FG, et al. Health problems of people with intellectual disabilities: the impact for general practice. *Br J Gen Pract* 2007;57(534):64-6. [published Online First: 2007/01/25]
43. Hayden MF, Kim SH, DePaepe P. Health status, utilization patterns, and outcomes of persons with intellectual disabilities: review of the literature. *Ment Retard*

For peer review only - http://bmjopen.bmj.com/site/about/guidelines.xhtml

2005;43(3):175-95. doi: 10.1352/0047-6765(2005)43[175:hsupao]2.0.co;2 [published Online First: 2005/05/11]

44. Ruddick L. Health of people with intellectual disabilities: a review of factors influencing access to health care. *British journal of health psychology* 2005;10(Pt 4):559-70. doi: 10.1348/135910705x43769 [published Online First: 2005/10/22]

45. Scheepers M, Kerr M, O'Hara D, et al. Reducing Health Disparity in People with Intellectual Disabilities: A Report from Health Issues Special Interest Research Group of the International Association for the Scientific Study of Intellectual Disabilities1. *Journal of Policy and Practice in Intellectual Disabilities* 2005;2(3-4):249-55. doi: 10.1111/j.1741-1130.2005.00037.x

46. Havercamp SM, Scandlin D, Roth M. Health disparities among adults with developmental disabilities, adults with other disabilities, and adults not reporting disability in North Carolina. *Public Health Rep* 2004;119(4):418-26. doi: 10.1016/j.phr.2004.05.006 [published Online First: 2004/06/29]

47. Havercamp SM, Scott HM. National health surveillance of adults with disabilities, adults with intellectual and developmental disabilities, and adults with no disabilities. *Disability and health journal* 2014 doi: 10.1016/j.dhjo.2014.11.002 [published Online First: 2015/01/18]

48. Ali A, Scior K, Ratti V, et al. Discrimination and other barriers to accessing health care: perspectives of patients with mild and moderate intellectual disability and their carers. *PloS one* 2013;8(8):e70855. doi: 10.1371/journal.pone.0070855 [published Online First: 2013/08/21]

49. Iacono T, Bigby C, Unsworth C, et al. A systematic review of hospital experiences of people with intellectual disability. *BMC Health Serv Res* 2014;14:505. doi: 10.1186/s12913-014-0505-5 [published Online First: 2014/10/26]

50. Sowney M, Barr O. The challenges for nurses communicating with and gaining valid consent from adults with intellectual disabilities within the accident and emergency care service. *J Clin Nurs* 2007;16(9):1678-86. doi: 10.1111/j.1365-2702.2006.01642.x [published Online First: 2007/04/27]

51. Llewellyn G, Vaughan C, Emerson E. Discrimination and the health of people with intellectual disabilities. International review of research in developmental disabilities: Elsevier 2015:43-72.

52. Axmon A, Sandberg M, Ahlstrom G. Gender differences in psychiatric diagnoses in older people with intellectual disability: a register study. *BMC psychiatry* 2017;17(1):192. doi: 10.1186/s12888-017-1353-8 [published Online First: 2017/05/24]

53. Hemm C, Dagnan D, Meyer TD. Identifying training needs for mainstream healthcare professionals, to prepare them for working with individuals with intellectual disabilities: a systematic review. *Journal of applied research in intellectual disabilities : JARID* 2015;28(2):98-110. doi: 10.1111/jar.12117 [published Online First: 2014/10/01]

54. Heslop P, Read S, Dunwoodie Stirton F. The hospital provision of reasonable adjustments for people with learning disabilities: Findings from Freedom of Information requests. *British Journal of Learning Disabilities* 2018;46(4):258-67. doi: 10.1111/bld.12244

55. Hosking FJ, Carey IM, DeWilde S, et al. Preventable Emergency Hospital Admissions Among Adults With Intellectual Disability in England. *The Annals of Family Medicine* 2017;15(5):462-70. doi: 10.1370/afm.2104

56. Ervin DA, Hennen B, Merrick J, et al. Healthcare for Persons with Intellectual and Developmental Disability in the Community. *Frontiers in public health* 2014;2(83) doi: 10.3389/fpubh.2014.00083

For peer review only - http://bmjopen.bmj.com/site/about/guidelines.xhtml

57. Trollor JN, Eagleson C, Turner B, et al. Intellectual disability health content within nursing curriculum: An audit of what our future nurses are taught. *Nurse Educ Today* 2016;45:72-9. doi: 10.1016/j.nedt.2016.06.011 [published Online First: 2016/07/19]
58. Trollor JN, Ruffell B, Tracy J, et al. Intellectual disability health content within medical curriculum: an audit of what our future doctors are taught. *BMC medical education* 2016;16:105. doi: 10.1186/s12909-016-0625-1 [published Online First: 2016/04/14]
59. Sullivan WF, Berg JM, Bradley E, et al. Primary care of adults with developmental disabilities: Canadian consensus guidelines. *Can Fam Physician* 2011;57(5):541-53, e154-68. [published Online First: 2011/05/17]
60. Carey IM, Shah SM, Hosking FJ, et al. Health characteristics and consultation patterns of people with intellectual disability: a cross-sectional database study in English general practice. *Br J Gen Pract* 2016;66(645):e264-e70.
61. Durbin A, Balogh R, Lin E, et al. Emergency Department Use: Common Presenting Issues and Continuity of Care for Individuals With and Without Intellectual and Developmental Disabilities. *J Autism Dev Disord* 2018;48(10):3542-50. doi: 10.1007/s10803-018-3615-9
62. Meijer M, Carpenter S, Scholte F. European manifesto on basic standards of health care for people with intellectual disabilities. *Journal of Policy and Practice in Intellectual Disabilities* 2004;1(1):10-15.
63. Scholte FA. European Manifesto: basic standards of healthcare for people with intellectual disabilities. *Salud Publica Mex* 2008;50 Suppl 2:s273-6. [published Online First: 2008/05/28]
64. Balogh R, McMorris CA, Lunsky Y, et al. Organising healthcare services for persons with an intellectual disability. *The Cochrane database of systematic reviews* 2016;4:Cd007492. doi: 10.1002/14651858.CD007492.pub2 [published Online First: 2016/04/12]

Figure legends:

Figure 1. Flowchart of the statistical analyses for somatic healthcare utilization (unplanned inpatient care, unplanned outpatient care, planned inpatient care, planned outpatient care) the following year.

Figure 2 ROC-curves for multivariate models predicting four types of somatic specialist healthcare utilization among 7936 older people with intellectual disability (ID), using data on age, sex, previous healthcare utilization, and relevant diagnoses.

For peer review only - http://bmjopen.bmj.com/site/about/guidelines.xhtml

Table 1 Relative risks (RRs) with 90% confidence intervals (CIs) of four types of somatic healthcare (unplanned/planned inpatient/outpatient) within a cohort of 7936 older people with intellectual disability (ID). All analyses are based on multivariate models adjusted for relevant diagnoses on ICD-10 block-level. Bold indicates statistically significantly difference (p<0.1) in RR.

	Unplanned		Planned	
	Inpatient	Outpatient	Inpatient	Outpatient
	RR (90% CI)	RR (90% CI)	RR (90% CI)	RR (90% CI)
Men vs women	1.04 (0.99-1.08)	**0.93 (0.88-0.99)**	**0.92 (0.85-0.99)**	**0.92 (0.88-0.95)**
**Age (years)**				
50-59 vs <50	**1.30 (1.18-1.44)**	1.12 (0.97-1.28)	**1.28 (1.05-1.55)**	**0.92 (0.86-0.99)**
60-69 vs <50	**1.50 (1.35-1.66)**	1.03 (0.90-1.19)	**1.48 (1.21-1.80)**	**0.79 (0.73-0.85)**
70-79 vs <50	**1.75 (1.57-1.96)**	0.91 (0.77-1.07)	**1.56 (1.26-1.93)**	**0.75 (0.69-0.82)**
80-89 vs <50	**2.07 (1.81-2.37)**	0.96 (0.75-1.24)	0.97 (0.69-1.39)	**0.54 (0.45-0.64)**
90+ vs <50	**2.33 (1.60-3.41)**	1.12 (0.51-2.45)		0.32 (0.08-1.24)
**Planned inpatient previous year**				
1 vs 0	**1.14 (1.04-1.24)**	1.05 (0.95-1.16)	**1.90 (1.64-2.21)**	1.00 (0.95-1.06)
2 vs 0	1.07 (0.89-1.29)	1.02 (0.83-1.26)	**2.89 (2.22-3.75)**	1.06 (0.94-1.20)
3+ vs 0	**1.43 (1.09-1.88)**	0.99 (0.68-1.45)	**4.03 (2.84-5.70)**	**1.34 (1.11-1.61)**
**Planned outpatient previous year**				
1 vs 0	**1.25 (1.19-1.33)**	**1.10 (1.02-1.18)**	**2.07 (1.86-2.30)**	**1.89 (1.80-1.99)**
2-5 vs 0	**1.40 (1.31-1.50)**	**1.34 (1.23-1.45)**	**2.77 (2.46-3.13)**	**2.59 (2.45-2.73)**
6-10 vs 0	1.23 (0.97-1.56)	0.94 (0.72-1.22)	**2.66 (1.83-3.87)**	**2.87 (2.59-3.18)**
11+ vs 0	1.36 (0.99-1.88)	**1.53 (1.11-2.11)**	**3.80 (2.51-5.76)**	**2.45 (2.05-2.93)**
**Unplanned inpatient previous year**				
1 vs 0	**2.02 (1.89-2.16)**	**1.14 (1.06-1.23)**	**1.59 (1.42-1.78)**	**1.04 (1.00-1.09)**
2 vs 0	**2.38 (2.17-2.62)**	**1.20 (1.08-1.34)**	**1.78 (1.49-2.14)**	**1.08 (1.00-1.16)**
3+ vs 0	**2.70 (2.39-3.04)**	**1.19 (1.05-1.35)**	**2.43 (2.03-2.90)**	**1.16 (1.07-1.26)**
**Unplanned outpatient previous year**				
1 vs 0	**1.25 (1.17-1.33)**	**1.78 (1.65-1.91)**	**1.20 (1.06-1.35)**	1.02 (0.97-1.07)
2-5 vs 0	**1.42 (1.29-1.56)**	**2.73 (2.47-3.02)**	**1.21 (1.00-1.46)**	**1.15 (1.08-1.23)**
6-10 vs 0	**1.33 (1.01-1.74)**	**3.66 (3.05-4.39)**	1.18 (0.69-2.00)	0.97 (0.78-1.20)
11+ vs 0	**1.52 (1.09-2.13)**	**3.74 (2.90-4.82)**	**2.22 (1.48-3.34)**	**1.45 (1.11-1.89)**

For peer review only

Table 2 Statistically significant (p<0.1) relative risks of four types of somatic healthcare (unplanned/planned inpatient/outpatient) within a cohort of 7936 older people with intellectual disability (ID). All analyses are based on multivariate models adjusted for sex, age, and healthcare utilization previous year.

	Unplanned		Planned	
	In-patient	Out-patient	In-patient	Out-patient
A20-A28: Certain zoonotic bacterial diseases				2.25
A30-A49: Other bacterial diseases				
A50-A64: Infections with a predominantly sexual mode of transmission	10.78			2.83
A90-A99: Arthropod-borne viral fevers and viral haemorrhagic fevers				1.45
B20-B24: Human immunodeficiency virus [HIV] disease	2.21			
B25-B34: Other viral diseases				
B50-B64: Protozoal diseases	4.29			
B95-B98: Bacterial, viral and other infectious agents		0.68		
B99-B99: Other infectious diseases				0.69
C00-C97: Malignant neoplasms	0.77			1.43
D00-D09: In situ neoplasms	2.18			
D80-D89: Certain disorders involving the immune mechanism				1.72
E10-E14: Diabetes mellitus	1.27	1.18		1.39
E15-E16: Other disorders of glucose regulation and pancreatic internal secretion		2.59		
E70-E90: Metabolic disorders	0.79	0.73		
F00-F09: Organic, including symptomatic, mental disorders				0.79
F10-F19: Mental and behavioural disorders due to psychoactive substance use	1.56	1.55		
F40-F48: Neurotic, stress-related and somatoform disorders		1.28		
F50-F59: Behavioural syndromes associated with physiological disturbances and physical factors	1.78			
F70-F79: Mental retardation		0.91		0.82
F80-F89: Disorders of psychological development		0.91		0.81
F99-F99: Unspecified mental disorder				
G20-G26: Extrapyramidal and movement disorders	1.32	1.38		1.36
G40-G47: Episodic and paroxysmal disorders	1.62	1.53		1.49
G60-G64: Polyneuropathies and other disorders of the peripheral nervous system	1.84			
G70-G73: Diseases of myoneural junction and muscle		1.93		
G80-G83: Cerebral palsy and other paralytic syndromes	1.31			
H15-H22: Disorders of sclera, cornea, iris and ciliary body		1.37		
H25-H28: Disorders of lens		0.81	1.37	
H30-H36: Disorders of choroid and retina				1.19
H40-H42: Glaucoma				1.61
H65-H75: Diseases of middle ear and mastoid				1.62
H90-H95: Other disorders of ear		1.37		

For peer review only - http://bmjopen.bmj.com/site/about/guidelines.xhtml

I10-I15: Hypertensive diseases				
I20-I25: Ischaemic heart diseases	1.17			1.12
I30-I52: Other forms of heart disease				1.17
I60-I69: Cerebrovascular diseases				
I80-I89: Diseases of veins, lymphatic vessels and lymph nodes, not elsewhere classified				0.83
J00-J06: Acute upper respiratory infections	1.28			0.77
J09-J18: Influenza and pneumonia				0.84
J20-J22: Other acute lower respiratory infections				
J30-J39: Other diseases of upper respiratory tract				1.28
J40-J47: Chronic lower respiratory diseases	1.19			
J60-J70: Lung diseases due to external agents				0.78
J95-J99: Other diseases of the respiratory system				
K40-K46: Hernia				
K50-K52: Noninfective enteritis and colitis		1.42		1.44
K55-K63: Other diseases of intestines	1.22	1.17	1.37	
K65-K67: Diseases of peritoneum				
K70-K77: Diseases of liver	1.35			1.46
L10-L14: Bullous disorders		2.70		
L20-L30: Dermatitis and eczema	1.46	1.66		
L50-L54: Urticaria and erythema	1.98			
L80-L99: Other disorders of the skin and subcutaneous tissue	0.64			
M00-M25: Arthropathies	1.13			1.22
M95-M99: Other disorders of the musculoskeletal system and connective tissue		2.39		
N00-N08: Glomerular diseases				1.51
N10-N16: Renal tubulo-interstitial diseases				0.72
N17-N19: Renal failure				1.16
N30-N39: Other diseases of urinary system	1.04			
N40-N51: Diseases of male genital organs	0.54			
P20-P29: Respiratory and cardiovascular disorders specific to the perinatal period	0.63			
Q20-Q28: Congenital malformations of the circulatory system	1.59			
Q60-Q64: Congenital malformations of the urinary system				1.50
Q90-Q99: Chromosomal abnormalities, not elsewhere classified		0.84		
R00-R09: Symptoms and signs involving the circulatory and respiratory systems	1.29	1.50		
R10-R19: Symptoms and signs involving the digestive system and abdomen	1.22	1.22		
R50-R69: General symptoms and signs				
S00-S09: Injuries to the head	1.16			
S30-S39: Injuries to the abdomen, lower back, lumbar spine and pelvis				
S60-S69: Injuries to the wrist and hand		1.18		
S80-S89: Injuries to the knee and lower leg		0.84		
T15-T19: Effects of foreign body entering through natural orifice	1.31			
T51-T65: Toxic effects of substances chiefly nonmedicinal as to source	2.00			
Z70-Z76: Persons encountering health services in other circumstances		1.29		

For peer review only - http://bmjopen.bmj.com/site/about/guidelines.xhtml

613

For peer review only

For peer review only - http://bmjopen.bmj.com/site/about/guidelines.xhtml

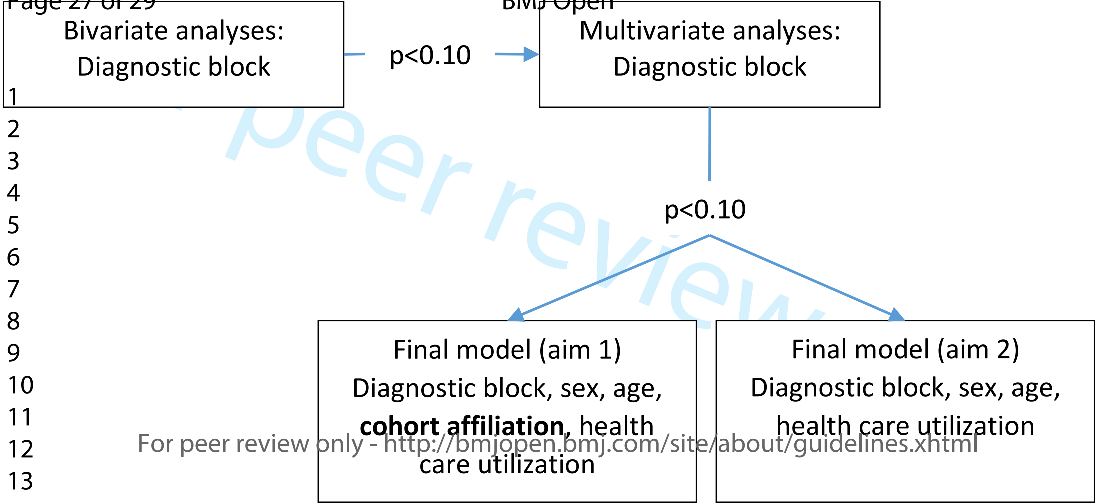

For peer review only - http://bmjopen.bmj.com/site/about/guidelines.xhtml

Unplanned inpatient care

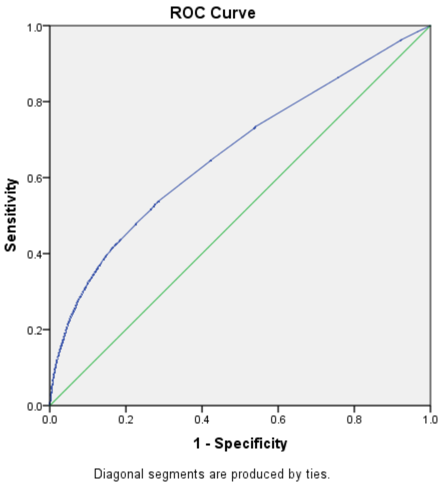

Unplanned outpatient care

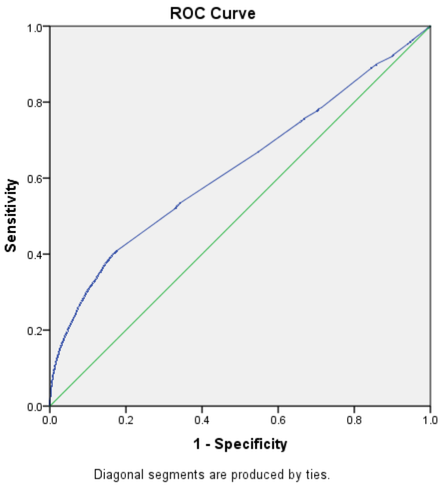

Planned inpatient care

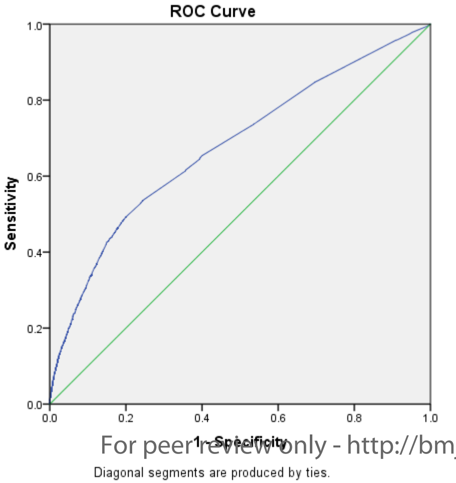

Planned outpatient care

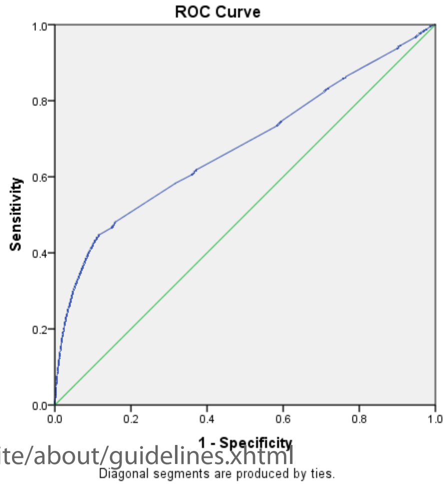

#1STROBE 2007 (v4) Statement—Checklist of items that should be included in reports of *cohort studies*

Section/Topic	Item #	Recommendation	Reported on page #
**Title and abstract**	1	(*a*) Indicate the study's design with a commonly used term in the title or the abstract	#1/Title page
		(*b*) Provide in the abstract an informative and balanced summary of what was done and what was found	#2-3
**Introduction**			
Background/rationale	2	Explain the scientific background and rationale for the investigation being reported	#6-7
Objectives	3	State specific objectives, including any prespecified hypotheses	#7
**Methods**			
Study design	4	Present key elements of study design early in the paper	#2 & #8
Setting	5	Describe the setting, locations, and relevant dates, including periods of recruitment, exposure, follow-up, and data collection	#8-9
Participants	6	(*a*) Give the eligibility criteria, and the sources and methods of selection of participants. Describe methods of follow-up	#8-9
		(*b*) For matched studies, give matching criteria and number of exposed and unexposed	#8-9
Variables	7	Clearly define all outcomes, exposures, predictors, potential confounders, and effect modifiers. Give diagnostic criteria, if applicable	#8-11
Data sources/ measurement	8*	For each variable of interest, give sources of data and details of methods of assessment (measurement). Describe comparability of assessment methods if there is more than one group	#9
Bias	9	Describe any efforts to address potential sources of bias	#9-11
Study size	10	Explain how the study size was arrived at	#8-9
Quantitative variables	11	Explain how quantitative variables were handled in the analyses. If applicable, describe which groupings were chosen and why	#9-11
Statistical methods	12	(*a*) Describe all statistical methods, including those used to control for confounding	#9-11
		(*b*) Describe any methods used to examine subgroups and interactions	#9
		(*c*) Explain how missing data were addressed	N/A
		(*d*) If applicable, explain how loss to follow-up was addressed	N/A
		(*e*) Describe any sensitivity analyses	N/A
**Results**			

For peer review only

Participants	13*	(a) Report numbers of individuals at each stage of study—eg numbers potentially eligible, examined for eligibility, confirmed eligible, included in the study, completing follow-up, and analysed	#9
		(b) Give reasons for non-participation at each stage	N/A
		(c) Consider use of a flow diagram	N/A
Descriptive data	14*	(a) Give characteristics of study participants (eg demographic, clinical, social) and information on exposures and potential confounders	N/A
		(b) Indicate number of participants with missing data for each variable of interest	N/A
		(c) Summarise follow-up time (eg, average and total amount)	N/A
Outcome data	15*	Report numbers of outcome events or summary measures over time	N/A
Main results	16	(*a*) Give unadjusted estimates and, if applicable, confounder-adjusted estimates and their precision (eg, 95% confidence interval). Make clear which confounders were adjusted for and why they were included	#11, Table 1, Table 2, Figure 2
		(*b*) Report category boundaries when continuous variables were categorized	#10
		(*c*) If relevant, consider translating estimates of relative risk into absolute risk for a meaningful time period	N/A
Other analyses	17	Report other analyses done—eg analyses of subgroups and interactions, and sensitivity analyses	N/A
**Discussion**			
Key results	18	Summarise key results with reference to study objectives	#13-15
**Limitations**			
Interpretation	20	Give a cautious overall interpretation of results considering objectives, limitations, multiplicity of analyses, results from similar studies, and other relevant evidence	#12-13
Generalisability	21	Discuss the generalisability (external validity) of the study results	
**Other information**			
Funding	22	Give the source of funding and the role of the funders for the present study and, if applicable, for the original study on which the present article is based	#3

*Give information separately for cases and controls in case-control studies and, if applicable, for exposed and unexposed groups in cohort and cross-sectional studies.

Note: An Explanation and Elaboration article discusses each checklist item and gives methodological background and published examples of transparent reporting. The STROBE checklist is best used in conjunction with this article (freely available on the Web sites of PLoS Medicine at http://www.plosmedicine.org/, Annals of Internal Medicine at http://www.annals.org/, and Epidemiology at http://www.epidem.com/). Information on the STROBE Initiative is available at www.strobe-statement.org.

For peer review only

BMJ Open

Predictors of specialist somatic healthcare utilization among older people with intellectual disability and their age-peers in the general population: A national register study

Journal:	*BMJ Open*
Manuscript ID	bmjopen-2023-072679.R1
Article Type:	Original research
Date Submitted by the Author:	16-May-2023
Complete List of Authors:	Sandberg, Magnus; Lunds Universitet, Department of Health Sciences<br>Axmon, Anna; Lunds University, EPI@LUND (Epidemiology, Population studies and Infrastructures at Lund University)<br>Ahlström, Gerd; Lunds Universitet, Health Sciences<br>Kristensson, Jimmie; Lunds Universitet, Department of Health Sciences; Lund University, Institute for Palliative Care
**Primary Subject Heading**:	Health services research
Secondary Subject Heading:	Epidemiology, Public health, Mental health
Keywords:	Organisation of health services < HEALTH SERVICES ADMINISTRATION & MANAGEMENT, Aged, REGISTRIES, Health Services Accessibility

SCHOLARONE™
Manuscripts

I, the Submitting Author has the right to grant and does grant on behalf of all authors of the Work (as defined in the below author licence), an exclusive licence and/or a non-exclusive licence for contributions from authors who are: i) UK Crown employees; ii) where BMJ has agreed a CC-BY licence shall apply, and/or iii) in accordance with the terms applicable for US Federal Government officers or employees acting as part of their official duties; on a worldwide, perpetual, irrevocable, royalty-free basis to BMJ Publishing Group Ltd ("BMJ") its licensees and where the relevant Journal is co-owned by BMJ to the co-owners of the Journal, to publish the Work in this journal and any other BMJ products and to exploit all rights, as set out in our licence.

The Submitting Author accepts and understands that any supply made under these terms is made by BMJ to the Submitting Author unless you are acting as an employee on behalf of your employer or a postgraduate student of an affiliated institution which is paying any applicable article publishing charge ("APC") for Open Access articles. Where the Submitting Author wishes to make the Work available on an Open Access basis (and intends to pay the relevant APC), the terms of reuse of such Open Access shall be governed by a Creative Commons licence – details of these licences and which Creative Commons licence will apply to this Work are set out in our licence referred to above.

Other than as permitted in any relevant BMJ Author's Self Archiving Policies, I confirm this Work has not been accepted for publication elsewhere, is not being considered for publication elsewhere and does not duplicate material already published. I confirm all authors consent to publication of this Work and authorise the granting of this licence.

For peer review only

Predictors of specialist somatic healthcare utilization among older people with intellectual disability and their age-peers in the general population: A national register study

Magnus Sandberg [a *], Anna Axmon [a, b], Gerd Ahlström [a], Jimmie Kristensson [a,c]

[a] Department of Health Sciences, Faculty of Medicine, Lund University, P.O. Box 157, Lund SE-221 00, Sweden

[b] EPI@LUND (Epidemiology, Population studies and Infrastructures at Lund University), Division of Occupational and Environmental Medicine, Lund University, Biskopsgatan 9, Lund SE-223 62, Sweden

c. Institute for Palliative Care, Lund University, Region Skåne

* Corresponding author:

Magnus Sandberg, Department of Health Sciences, Faculty of Medicine, Lund University, P.O. Box 157, SE-221 00 Lund, Sweden E-mail: Magnus.Sandberg@med.lu.se. Tel.: +46 46 222 19 71

For peer review only

Abstract

Objectives: To compare somatic healthcare utilization among older people with intellectual disabilities (ID) to that of their age-peers in the general population, taking into account health and demographic factors, and to identify predictors for somatic healthcare utilization among older people with ID.

Participants: Equally sized cohorts, one with people with ID and one referent cohort, one-to-one-matched by sex and year of birth, were created. Each cohort comprised 7936 people aged 55+ years at the end of 2012.

Design: Retrospective register-based study.

Setting: All specialist in- and outpatient healthcare clinics in Sweden.

Outcome measures: Data regarding planned/unplanned and inpatient/outpatient specialist healthcare were collected from the Swedish National Patient Register for 2002-2012. Diagnoses, previous healthcare utilization, sex, age, and cohort affiliation was used to investigate potential impact on healthcare utilization.

Results: Compared to the referent cohort, the ID cohort were more likely to have unplanned inpatient and outpatient care but less likely to have planned outpatient care. Within the ID cohort, sex, age and previous use of healthcare predicted healthcare utilization.

Conclusions: Older people with ID seem to have lower risks of planned outpatient care compared to the general population that could not be explained by diagnoses. Potential explanations are that people with ID suffer from communication difficulties and experience the healthcare environment as unfriendly. Moreover, that healthcare staff lack knowledge about the particular needs of people with ID. Altogether, this may lead to people with ID being exposed to discrimination. Although these problems are known, few interventions have been evaluated, especially related to planned outpatient care.

For peer review only - http://bmjopen.bmj.com/site/about/guidelines.xhtml

Keywords: healthcare utilisation, health services research, intellectual disability, older adults, Register studies

Strengths and limitations of this study

- A strength is that the study uses data from 11 consecutive years allowing analyses to investigate healthcare utilization following specific diagnoses adjusted for previous healthcare utilization.
- Another strength is that the study uses national registers with high validity data.
- A limitation of the study is that primary healthcare data are not included.
- Another limitation is that both the ID and the referent cohort consist of healthy survivors.

Word count

3500 (excluding the title page, abstract, tables, acknowledgements, authors' contributions, and references)

For peer review only

Background

A range of chronic diseases are found to a higher degree among people with intellectual disability (ID) than in the general population. These include diabetes [1 2], cardiovascular diseases [3], osteoporosis [4], visual and hearing impairment [5 6], musculoskeletal diseases [6], and thyroid conditions [5].

Recent decades have seen an increasing life span among people with ID, resulting in an increasing number of older people with ID [7]. A consequence of this is that people with ID live longer with chronic diseases, and it is well known that older people with ID experience high rates of multi-morbidity [8 9]. Thus, older people with ID would be expected to have greater healthcare needs and utilization than their age-peers in the general population. Even so, they have been found to have lower rates of specialist healthcare, especially in higher ages [10]. A possible explanation for this is that healthcare is not necessarily sought at the right care level, i.e., whereas older people in the general population are often referred to specialist care, older people with ID are treated in primary care. Another reason may be that some disorders are underdiagnosed/treated among older people with ID. For example, many pain and psychiatric diagnoses depend on the ability of the patient to communicate symptoms, which may be difficult for people of more severe ID [11 12].

Previous studies have reported that older people in the general population with physical as well as social unmet needs are often admitted to acute medical wards [13 14]. Thus, having a high amount of unplanned care and low of planned care could be an indication of unmet needs. To enable provision of proper healthcare to older people with ID, it is important to investigate factors that may predict different forms of healthcare utilization.

Previous studies regarding somatic healthcare utilization among people with ID have demonstrated associations between a range of demographic (e.g. age, disability level), socioeconomic (e.g. income, living arrangements), health (e.g. subjective and self-perceived

For peer review only

For peer review only - http://bmjopen.bmj.com/site/about/guidelines.xhtml

health status), and treatment (e.g. polypharmacy) factors on one hand, and hospital admissions [15-19] and outpatient care [20-22] on the other. There are also studies investigating various healthcare utilization patterns [23 24]. However, the majority of these studies are cross-sectional without the possibility to investigate predictors of healthcare utilization. In addition, they investigate only subgroups of the ID population, for example those at day care centres [16], admitted for orthopaedic procedures [23] or enrolled in a home and community-based waiver [20]. In one study [17] that included both in- and outpatient data, outpatient visits was only measured with a dichotomised variable, more than 24 visits, or 24 or less visits. One exception is a comprehensive English study [24] investigating emergency department admissions and annual health check among 21 859 adults with ID compared with 152 846 age-, gender- and practice-matched controls without ID in 2009–2013. They found that people with ID were more likely than the general population to have emergency department admissions, and for reasons that were more likely to be deemed as preventable also after adjusting for nine different comorbidities. They also found that annual health checks for adults with ID did not have any effects on emergency department admissions compared to controls [24]. But also here, they only investigated certain healthcare types and diagnoses. Hence, there is a need for studies investigating somatic healthcare utilization over a longer period.

The aims of the present study were to 1) compare somatic specialist healthcare utilization among older people with ID to that of their age-peers in the general population, taking into account a range of health and demographic variables and 2) develop a model for prediction of healthcare utilization among older people with ID.

For peer review only - http://bmjopen.bmj.com/site/about/guidelines.xhtml

Material and Methods

Study design

This was a longitudinal, retrospective, population register-based study. The study was approved by the Regional Ethical Review Board in Lund (diary no 2013/15), and performed in accordance with the Helsinki declaration [25].

Setting

Sweden has a welfare system mainly funded by taxes [26]. People with permanent functional impairments can receive support from the municipality according to the Act concerning Support and Service for Persons with Certain Functional Impairments (LSS) [27]. The support available is regulated in the LSS act, and includes e.g., daytime activities and residential care. It also includes support to next of kin, for example relief services. All provided support is recorded in the national LSS-register, which is maintained by the Swedish National Board of Health and Welfare. Reporting to this register is mandatory for all municipalities. The LSS act distinguishes between three types of functional impairments, whereof one is those that have been present since birth or early years. These comprise ID and autism spectrum disorders (ASD). Thus, it is possible to separate this group of people from those with intellectual functional impairment acquired in adult life, for example caused by traumatic brain injury. People who cannot manage their day-to-day existence, have the possibility to apply for personal assistance or municipal care and/or social services according to the Social Services Act [28]. However, in Sweden, as in many other countries, informal caregivers are responsible for much of the care.

Specialist healthcare in outpatient and inpatient facilities, primary care, rehabilitation and advanced home care provided by the 21 county councils is regulated by the Health and

For peer review only - http://bmjopen.bmj.com/site/about/guidelines.xhtml

Medical Services Act (HSL) [29]. The act establishes equal access to healthcare for all Swedish residents regardless of where they live. Specialist inpatient and outpatient healthcare provided according to the HSL is registered in the National Patient Register (NPR). This register contains information on inpatient episodes and outpatient visits, with one primary and up to 21 secondary diagnoses recorded according to ICD-10 (International Statistical Classification of Diseases and Related Health Problems 10th Revision) [30] at discharge. Data are also available on whether the healthcare episode was planned (i.e., if an appointment or admission was made beforehand) or unplanned. Unplanned episodes are more or less acute, and can be everything from an emergency department visit (unplanned outpatient visit) to an unplanned admission to an orthopaedic ward because of gallstone (unplanned inpatient care). The dates of hospital admission and discharge are recorded for inpatient healthcare, and the date of the visit for outpatient specialist healthcare. In this study, only data from somatic healthcare were used, but nonetheless, these could include both somatic and psychiatric diagnoses.

Study populations

The ID cohort comprised all people aged 55 years and older who received support and social services according to the LSS act [27] during 2012, were recorded as belonging to the group of people with functional impairment since birth or early age (i.e. having ID and/or ASD), and alive at the end of 2012. The cut-off at 55 years was chosen to define “old” because the aging process can start earlier in people with ID [31]. A referent cohort from the general population (gPop cohort) was established through one-to-one matching by birth year and sex from the Swedish National Population Register [32]. The matching was performed by Statistics Sweden [33]. In total, 15 872 people were included in the study, 7936 in the ID cohort and an equal number in the gPop cohort. In both groups the mean age in 2012 was 65 years and 45.5% were women. However, as the data was collected retrospectively with respect to

For peer review only - http://bmjopen.bmj.com/site/about/guidelines.xhtml

the sample, their age at the beginning of the study period in 2002 was 44 or older. If a person was included in the ID cohort, they could not be matched as a referent.

Material

Pseudonymized in- and outpatient healthcare data for all individuals in the two cohorts were collected from the NPR for the 11-year period from January 1, 2002, to December 31, 2012. An individual could have several registrations for one inpatient period because of a change in ward during the hospital stay. In this paper, data on all registrations and their corresponding diagnoses are used.

Patient and public involvement

Patients and/or the public were not involved in the design, or conduct, or reporting or dissemination plans of this research. However, representatives from a patient organisation FUB, The Swedish National Association for People with Intellectual Disability, which is an advocacy organization working to enable people with an ID to live a good life, has been included in the project's reference group.

Statistical analysis

Potential impact of diagnoses, previous healthcare utilization, sex, age, and cohort affiliation (aim 1 only) on the four types of healthcare utilization (planned/unplanned, inpatient/outpatient healthcare) was evaluated using Poisson models, thus estimating relative risks (RRs) with 95% confidence intervals (CI). Calendar year was used to indicate repeated measures. A similar method was employed to evaluate aim 2, with the exception that only data from the ID cohort were used and thus cohort affiliation was not included in any of the models.

For peer review only - http://bmjopen.bmj.com/site/about/guidelines.xhtml

Diagnoses were investigated on block level, as defined in ICD-10. Yearly inpatient healthcare utilization was categorized as 0, 1, 2, or 3+ visits, whereas yearly outpatient healthcare utilization was categorized as 0, 1, 2-5, 6-10, or 11+ visits. Age was aggregated to 10-year intervals.

Both aims and all four types of healthcare utilization were investigated using similar procedures (Figure 1): First, bivariate analyses were performed to investigate possible associations between different diagnoses and healthcare utilization. Second, for each type of healthcare and aim, a multivariate model was employed, including all diagnoses with $p<0.10$ in the bivariate models. Third, all diagnoses with $p<0.10$ in the multivariate model were included in final models (one per type of healthcare and aim) which also included cohort affiliation (aim 1 only), sex, age, and healthcare utilization the previous year. Fourth, the predictive ability of two different models was assessed using the Area Under the Curve (AUC).

In the final model for planned inpatient care in the ID cohort, the Hessian matrix was singular and the convergence criteria were not satisfied. We therefore ran collinearity diagnostics. This was done in a plain regression model as the GML module in SPSS does not provide this option. Diagnostic chapters were removed from the final model one by one according to their VIF-value until the convergence criteria were satisfied. Hence, these blocks were removed due to multicollinearity: A50-A64 (Infections with a predominantly sexual mode of transmission), P10-P15 (Birth trauma), Q60-Q64 (Congenital malformations of the urinary system), B50-B64 (Protozoal diseases), G50-G59 (Nerve, nerve root and plexus disorders), K70-K77 (Diseases of liver), D00-D09 (In situ neoplasms), K65-K67 (Diseases of peritoneum), G20-G26 (Extrapyramidal and movement disorders), J30-J39 (Other diseases of upper respiratory tract), I80-I89 (Diseases of veins, lymphatic vessels and lymph nodes, not elsewhere classified), K50-K52 (Noninfective enteritis and colitis), J95-J99 (Other diseases of

For peer review only

the respiratory system), M80-M94 (Osteopathies and chondropathies), R30-R39 (Symptoms and signs involving the urinary system), K80-K87 (Disorders of gallbladder, biliary tract and pancreas), K40-K46 (Hernia), G80-G83 (Cerebral palsy and other paralytic syndromes), F70-F79 (Mental retardation), M60-M79 (Soft tissue disorders), I20-I25 (Ischemic heart diseases), M00-M25 (Arthropathies), and Z40-Z54 (Persons encountering health services for specific procedures and healthcare).

All analyses were made in IBM SPSS statistics Version 23.

<<Insert Figure 1 about here>>

Results

In multivariate models including both cohorts, people in the ID cohort had increased risk of unplanned care – both inpatient and outpatient – compared to the gPop cohort (RR 1.40; 95% CI 1.33-1.48, and RR 1.14; 95% CI 1.08-1.20, respectively). However, those with ID had decreased risk of planned outpatient care (RR 0.91; 95% CI 0.89-0.94). There was no change in risk for planned inpatient care (RR 1.01; 95% CI 0.94-1.09).

Within the ID cohort, men had decreased risk of unplanned outpatient care as well as planned care (both inpatient and outpatient) compared to women (RR 0.93, 95% CI 0.88-0.99, RR 0.92; 95% CI 0.85-0.99, and RR 0.92; 95% CI 0.88-0.95 respectively; Table 1). Increasing age was associated with increased risk of inpatient care, both planned and unplanned, but with decreased risk of planned outpatient care (Table 1). Again, with only a few exceptions, healthcare the previous year carried an increased risk of healthcare utilization (Table 1). No diagnosis increased the risk of any type of healthcare (Table 2).

All models, i.e. based on ID cohort only, and for all four types of care, were able to predict healthcare utilization better than chance (Figure 2).

For peer review only

For peer review only - http://bmjopen.bmj.com/site/about/guidelines.xhtml

<<Insert Table 1 about here>>

<<Insert Table 2 about here>>

<<Insert Figure 2 about here>>

Discussion

Even when adjusting for demographic factors, all recorded diagnoses, and previous healthcare utilization, ID was associated with increased unplanned healthcare utilization. If unplanned healthcare is a measure of unmet healthcare needs, this is cause for concern. Another important finding was the lower healthcare utilization among men compared to women among people with ID, even when all recorded diagnoses were taken into account. This suggests gender differences in access to healthcare among older people with ID.

The major strength of the present study is the use of national registers with high validity to identify people with ID as well as healthcare utilization and diagnoses. In using data from 11 consecutive years and repeated measures analyses, we were able to investigate healthcare utilization following specific diagnoses, rather than mere associations (i.e. non-directional), as well as adjust for previous healthcare utilization. Thus, the results presented for demographic factors and diagnoses are most likely not inflated by people with particularly high healthcare consumption. In addition, reporting of inpatient- and specialist outpatient care to NPR is mandatory for healthcare providers and the register has been shown to have high validity [34].

A potential weakness with the present study is the use of an administrative ID cohort. The LSS register comprises all people who have received service and support aimed at people with ID and/or ASD, including support to informal caregivers. However, those who did not

For peer review only - http://bmjopen.bmj.com/site/about/guidelines.xhtml

receive such support in 2012 were not included in the ID cohort. This may include, for example, people with ID who are being taken care of solely by parents or other family without support from the municipality. Even so, the Swedish welfare system is designed so that vulnerable groups are cared for by the state, county, or municipality. It is rare that informal caregivers within the family do not receive any support at all. In addition, considering the age group studied, we believe that the number of people with ID being cared for by their parents is low. Another limitation with the LSS register is that the level of ID is not registered. We have therefore not been able to take this into account. We have previously used level of ID registered in the NPR, however such analyses excluded those without such a diagnosis during the study period, including those without any healthcare utilization [35]. As the data were collected retrospectively for people alive at the end of the study period, another potential weakness could be cohort effects, i.e., that people with ID that survived and turned 55 years or older might be healthier than their younger non-surviving peers.

We collected information on healthcare utilization as well as diagnoses from the NPR. Although this register has national coverage of specialist care, it does not include visits to primary care. Thus, even though we have good coverage of specialist healthcare utilization, it is difficult to interpret the results in terms of unmet *overall* healthcare needs. As people with ID are less likely than those in the general population to get referrals from primary to specialist care [36] any bias introduced from the lack of primary healthcare data should have caused an underestimation of the higher healthcare utilization associated with having ID. In addition, the diagnoses are aggregated over an 11-year period and no consideration was taken to multi-morbidity. To get a deeper understanding of the determinants of unplanned healthcare and the adequacy of the care, a limited number of diagnoses, or combinations of diagnoses, should be investigated more in detail in future studies.

For peer review only - http://bmjopen.bmj.com/site/about/guidelines.xhtml

One of our main findings is that people with ID have less planned outpatient care than the general population, with lower risks in higher ages, which is not explained by differences in diagnoses. This is surprising as people with ID have a more complex disease panorama than the general population [4-6 9], and as the prevalence of multiple chronic diseases is a well-known explanation for higher amount of healthcare utilization in the general older population [37]. In addition, it is reasonable to assume that unplanned care in terms of hospital admissions are followed up with planned forms of care and services in order to prevent readmission [38]. Previous research has also found that annual health checks seem to reduce preventable emergency admissions [24]. Only a few studies have investigated healthcare use among people with ID compared to that in the general population [39-44]. Of these, only Skorpen, et al. [43] reported results consistent with the present study, with adults with ID being hospitalised more frequently at younger age and less frequently at older ages compared to the general population. Most studies did not consider the influence of age on healthcare utilization [39 40 42 44]. However, Hsu, et al. [41] investigated ambulatory care visits among older people (65 years and older) with and without ID, and found that people with ID had a higher mean number of visits and mean annual visits, and that age group affected the annual visits with middle-elderly (74-84) in both groups having the highest frequency of visits. There is a range of possible explanations for the lower rates of planned healthcare among people with ID. These include difficulties accessing the healthcare system [45-47] and lower rates of planned preventive healthcare, such as breast and cervical cancer screening [48 49]. Furthermore, even when having entered the healthcare system, people with ID may have communication difficulties and experience the healthcare environment as unfriendly [50-52]. An explanation of particular concern is that the ID population is discriminated by the healthcare system [53].

In addition to being discriminated in relation to the general population, there could also be discrimination between different groups within the ID population. The present study also

For peer review only - http://bmjopen.bmj.com/site/about/guidelines.xhtml

revealed that women with ID have higher healthcare utilization than men with ID. Gender differences in psychiatric diagnoses have been reported [54], but do not seem to have been investigated when it comes to somatic healthcare utilization. Thus, it is important to investigate whether males with ID are at risk for a “double discrimination”.

Even though the need for interventions regarding requirement of knowledge about and various aspects of supporting people with ID have been addressed since the 1990’ies [55], there is a lack of interventions studies and the reduced access to healthcare among people with ID still persists today [50 56]. In addition, adults with ID have been reported to be at high risk for preventable unplanned admissions, despite having similar primary care utilization before admission as the general population [57]. Hence, further interventions are needed, e.g. such that focus on communication, knowledge/information and profession-specific needs [55]. This may include enhancements of Medical- and Nursing Schools curriculums [58-60] to increase knowledge about people with ID, which may facilitate access to healthcare, which in turn may support the health and wellbeing among people with ID. Moreover, interventions aimed at primary care providers may be relevant, as these often are the gate keepers to other forms of care and often have continuous contact with the person with ID, and thus, have the possibility to coordinate care, and also to provide disease prevention, early detection, and appropriate management [61]. People with ID in primary care have been reported to have lower continuity of care and be less likely to have longer appointments than the general population, which could be areas of improvement [62], especially as higher continuity of care has been found to be associated to emergency department visits among people with ID [63]. Even though initiatives have been taken to increase the number of people with ID that seek primary healthcare [58 64 65], the effects of these interventions do not seem to have been evaluated. Furthermore, few studies have evaluated continuity of care interventions for people with ID and none with

For peer review only

For peer review only - http://bmjopen.bmj.com/site/about/guidelines.xhtml

unplanned admissions as primary outcome [66]. Thus, this needs to be addressed in future research.

Conclusions

People with ID have less planned outpatient healthcare than the general population. This difference was not explained by diagnoses, demographic factors, or previous healthcare utilization. Thus, it is likely a result of other factors, for example communication difficulties and experiences of the healthcare environment as unfriendly among people with ID, as well as a lack of knowledge about ID among healthcare staff, and discrimination of people with ID. Discrimination could also be the reason for less planned and more unplanned inpatient and outpatient specialist care among those with ID. These problems have been addressed for a long time, but there are still few intervention studies in the area and especially studies targeting the problems related to planned healthcare identified in the present study. Thus, there is a great need for evaluation of healthcare interventions, especially in planned outpatient care such as primary care, which aim to give people with ID access to healthcare to the same extent as the general population.

Acknowledgements

We would like to acknowledge FORTE – The Swedish Research Council for Health, Working Life and Welfare (grant no. 2014–4753) and the Faculty of Medicine, Lund University, Sweden for financing this study.

Ethics statements

Patient consent for publication

For peer review only - http://bmjopen.bmj.com/site/about/guidelines.xhtml

Not applicable.

Ethics approval

Approval was obtained from the Regional Ethical Review Board in Lund (reg no 2013/15). The Swedish National Board of Health and Welfare as well as Statistics Sweden performed a separate secrecy review in 2014 before providing access to the data. All analyses were performed using pseudonymized datasets.

Funding statement

This work was funded by Forte, the Swedish Research Council for Health, Working Life and Welfare (http://forte.se/en) no. 2014-4753 with GA as PI. The funders had no role in study design, data collection and analysis, decision to publish, or preparation of the manuscript.

Data sharing statement

The data in the present study contains sensitive information on a very vulnerable group, i.e., people with ID. However, as our database is compiled by register data only, other researchers may contact Statistics Sweden and the Swedish National Board of Health and Welfare to get access to the different registers included, and thereby recreate the data

Author contributions

MS participated in the design of the study and in the collection of data, interpretation of data, and drafted the manuscript. GA participated in the design of the study and helped in the interpretation of the data and drafting of the manuscript. AA participated in the design of the

For peer review only

For peer review only - http://bmjopen.bmj.com/site/about/guidelines.xhtml

study, performed the analysis, interpretation of data and helped in the drafting of the manuscript. JK participated in the design of the study and helped in the interpretation of the data and drafting the manuscript. All authors read and approved the final manuscript.

Conflict of Interests Statements

The authors have declared that no competing interests exist.

For peer review only

For peer review only - http://bmjopen.bmj.com/site/about/guidelines.xhtml

References

1. Axmon A, Ahlstrom G, Hoglund P. Prevalence and treatment of diabetes mellitus and hypertension among older adults with intellectual disability in comparison with the general population. *BMC Geriatr* 2017;17(1):272. doi: 10.1186/s12877-017-0658-2 [published Online First: 2017/11/25]
2. Flygare Wallen E, Ljunggren G, Carlsson AC, et al. High prevalence of diabetes mellitus, hypertension and obesity among persons with a recorded diagnosis of intellectual disability or autism spectrum disorder. *J Intellect Disabil Res* 2018;62(4):269-80. doi: 10.1111/jir.12462 [published Online First: 2017/12/28]
3. Draheim CC. Cardiovascular disease prevalence and risk factors of persons with mental retardation. *Mental retardation and developmental disabilities research reviews* 2006;12(1):3-12.
4. van Schrojenstein Lantman-De Valk HM, Van Den Akker M, Maaskant MA, et al. Prevalence and incidence of health problems in people with intellectual disability. *J Intellect Disabil Res* 1997;41(1):42-51.
5. Kapell D, Nightingale B, Rodriguez A, et al. Prevalence of chronic medical conditions in adults with mental retardation: comparison with the general population. *Ment Retard* 1998;36(4):269-79. doi: 10.1352/0047-6765(1998)036<0269:pocmci>2.0.co;2 [published Online First: 1998/08/26]
6. van Schrojenstein Lantman-De Valk HM, Metsemakers JF, Haveman MJ, et al. Health problems in people with intellectual disability in general practice: a comparative study. *Fam Pract* 2000;17(5):405-7. [published Online First: 2000/10/06]
7. Fisher K, Kettl P. Aging with mental retardation: increasing population of older adults with MR require health interventions and prevention strategies. *Geriatrics* 2005;60(4):26-29.
8. McCarron M, Swinburne J, Burke E, et al. Patterns of multimorbidity in an older population of persons with an intellectual disability: results from the intellectual disability supplement to the Irish longitudinal study on aging (IDS-TILDA). *Res Dev Disabil* 2013;34(1):521-7. doi: 10.1016/j.ridd.2012.07.029 [published Online First: 2012/10/23]
9. Sandberg M, Ahlstrom G, Kristensson J. Patterns of Somatic Diagnoses in Older People with Intellectual Disability: A Swedish Eleven Year Case-Control Study of Inpatient Data. *Journal of applied research in intellectual disabilities : JARID* 2017;30(1):157-71. doi: 10.1111/jar.12230 [published Online First: 2015/11/07]
10. Sandberg M, Ahlstrom G, Axmon A, et al. Somatic healthcare utilisation patterns among older people with intellectual disability: an 11-year register study. *BMC Health Serv Res* 2016;16(1):642. doi: 10.1186/s12913-016-1880-x [published Online First: 2016/11/11]
11. Axmon A, Ahlström G, Westergren H. Pain and Pain Medication among Older People with Intellectual Disabilities in Comparison with the General Population. *Healthcare* 2018;6(2):67.
12. Boardman L, Bernal J, Hollins S. Communicating with people with intellectual disabilities: a guide for general psychiatrists. *Advances in psychiatric treatment* 2014;20(1):27-36.
13. Campbell SE, Seymour DG, Primrose WR. A systematic literature review of factors affecting outcome in older medical patients admitted to hospital. *Age Ageing* 2004;33(2):110-5. doi: 10.1093/ageing/afh036 [published Online First: 2004/02/13]

For peer review only

For peer review only - http://bmjopen.bmj.com/site/about/guidelines.xhtml

14. Molloy GJ, McGee HM, O'Neill D, et al. Loneliness and emergency and planned hospitalizations in a community sample of older adults. *J Am Geriatr Soc* 2010;58(8):1538-41. doi: 10.1111/j.1532-5415.2010.02960.x [published Online First: 2010/07/22]

15. Lin JD, Loh CH, Yen CF, et al. Medical care services for people with intellectual disabilities living in the general community: a cross-sectional survey of inpatient care utilization in Taiwan, 2001. *Disabil Rehabil* 2007;29(18):1411-6. doi: 10.1080/09638280600999576 [published Online First: 2007/08/31]

16. Lin JD, Wu JL, Lee PN. Utilization of inpatient care and its determinants among persons with intellectual disabilities in day care centres in Taiwan. *J Intellect Disabil Res* 2004;48(Pt 7):655-62. doi: 10.1111/j.1365-2788.2003.00572.x [published Online First: 2004/09/11]

17. Loh CH, Lin JD, Choi IC, et al. Longitudinal analysis of inpatient care utilization among people with intellectual disabilities: 1999-2002. *J Intellect Disabil Res* 2007;51(Pt 2):101-8. doi: 10.1111/j.1365-2788.2006.00822.x [published Online First: 2007/01/16]

18. Merrick J, Davidson PW, Morad M, et al. Older adults with intellectual disability in residential care centers in Israel: health status and service utilization. *Am J Ment Retard* 2004;109(5):413-20. doi: 10.1352/0895-8017(2004)109<413:oawidi>2.0.co;2 [published Online First: 2004/08/10]

19. Venkat A, Pastin RB, Hegde GG, et al. An analysis of ED utilization by adults with intellectual disability. *Am J Emerg Med* 2011;29(4):401-11. doi: 10.1016/j.ajem.2009.11.009 [published Online First: 2010/09/10]

20. Hall A, Wood D, Hou T, et al. Patterns in primary health care utilization among individuals with intellectual and developmental disabilities in Florida. *Intellectual and developmental disabilities* 2007;45(5):310-22. doi: 10.1352/0047-6765(2007)45[310:piphcu]2.0.co;2 [published Online First: 2007/09/25]

21. Lin JD, Wu JL, Lee PN. Healthcare needs of people with intellectual disability in institutions in Taiwan: outpatient care utilization and implications. *J Intellect Disabil Res* 2003;47(Pt 3):169-80. [published Online First: 2003/02/27]

22. Lin JD, Yen CF, Loh CH, et al. A cross-sectional study of the characteristics and determinants of emergency care utilization among people with intellectual disabilities in Taiwan. *Res Dev Disabil* 2006;27(6):657-67. doi: 10.1016/j.ridd.2005.09.001 [published Online First: 2005/11/10]

23. Bathje M, Conrad S, Medick M, et al. Differences in Hospital-Based Care for Patients With Intellectual and Developmental Disabilities. *Am J Occup Ther* 2021;75(3) doi: 10.5014/ajot.2021.046508

24. Carey IM, Hosking FJ, Harris T, et al. Health Services and Delivery Research. An evaluation of the effectiveness of annual health checks and quality of health care for adults with intellectual disability: an observational study using a primary care database. Southampton (UK): NIHR Journals Library

25. World Medical Association. World medical association declaration of helsinki: Ethical principles for medical research involving human subjects. *JAMA* 2013;310(20):2191-94. doi: 10.1001/jama.2013.281053

26. Anell A, Glenngard AH, Merkur SM. Sweden: Health system review. *Health systems in transition* 2012;14(5):1-159.

27. *Lag om stöd och service till vissa funktionshindrade (LSS) [Act concerning Support and Service for Persons with Certain Functional Impairments]*. SFS 1993:387. Stockholm: Socialdepartementet. Available: https://www.riksdagen.se/sv/dokument-

For peer review only

For peer review only - http://bmjopen.bmj.com/site/about/guidelines.xhtml

lagar/dokument/svensk-forfattningssamling/lag-1993387-om-stod-och-service-till-vissa_sfs-1993-387 [Accessed 08 Feb 2023].

28. *Socialtjänstlagen [The Social Services Act*]. SFS 2001:453. . Stockholm: Socialdepartementet. Available: https://www.riksdagen.se/sv/dokument-lagar/dokument/svensk-forfattningssamling/socialtjanstlag-2001453_sfs-2001-453. [Accessed 12 May 2023].

29. *Hälso- och sjukvårdslag* [*The Health and Medical Service Act*]. SFS 2017:30. Stockholm: Socialdepartementet. Available: https://www.riksdagen.se/sv/dokument-lagar/dokument/svensk-forfattningssamling/halso--och-sjukvardslag_sfs-2017-30 [Accessed 08 Feb 2023].

30. World Health Organisation. International Statistical Classification of Diseases and Related Health Problems, 10th Revision, 2015 Version Geneva2015 [Available from: https://icd.who.int/browse10/2019/en#/ [Accessed 08 Feb 2023].

31. Thorpe L, Davidson PW, Janicki MP. Healthy Ageing – Adults with Intellectual Disabilities: Biobehavioural Issues. *Journal of Applied Research in Intellectual Disabilities* 2001;14:218-28.

32. Wilén A, Johannesson I. A new Total population register system: More possibilities and better quality. Örebro, 2002.

33. Statistics Sweden. Statistic Sweden startpage 2019 [Available from: http://www.scb.se/en_/ [Accessed 08 Feb 2023].

34. Ludvigsson JF, Andersson E, Ekbom A, et al. External review and validation of the Swedish national inpatient register. *BMC Public Health* 2011;11:450. doi: 1471-2458-11-450 [published Online First: 2011/06/11]

35. Axmon A, Björne P, Nylander L, et al. Psychiatric diagnoses in relation to severity of intellectual disability and challenging behaviors: a register study among older people. *Aging Ment Health* 2018;22(10):1344-50. doi: 10.1080/13607863.2017.1348483 [published Online First: 20170821]

36. Weise J, Pollack A, Britt H, et al. Primary health care for people with an intellectual disability: an exploration of consultations, problems identified, and their management in Australia. *J Intellect Disabil Res* 2017;61(5):399-410. doi: 10.1111/jir.12352 [published Online First: 2017/01/25]

37. Lehnert T, Heider D, Leicht H, et al. Review: health care utilization and costs of elderly persons with multiple chronic conditions. *Med Care Res Rev* 2011;68(4):387-420. doi: 10.1177/1077558711399580 [published Online First: 2011/08/05]

38. Feigenbaum P, Neuwirth E, Trowbridge L, et al. Factors contributing to all-cause 30-day readmissions: a structured case series across 18 hospitals. *Med Care* 2012:599-605.

39. Balogh RS, Hunter D, Ouellette-Kuntz H. Hospital Utilization among Persons with an Intellectual Disability, Ontario, Canada, 1995–2001. *Journal of Applied Research in Intellectual Disabilities* 2005;18(2):181-90. doi: 10.1111/j.1468-3148.2005.00247.x

40. Beange H, McElduff A, Baker W. Medical disorders of adults with mental retardation: a population study. *Am J Ment Retard* 1995;99(6):595-604. [published Online First: 1995/05/01]

41. Hsu SW, Lin JD, Chiang PH, et al. Comparison of outpatient services between elderly people with intellectual disabilities and the general elderly population in Taiwan. *Res Dev Disabil* 2012;33(5):1429-36. doi: 10.1016/j.ridd.2012.03.014 [published Online First: 2012/04/24]

42. Lunsky Y, Lin E, Balogh R, et al. Are adults with developmental disabilities more likely to visit EDs? *Am J Emerg Med* 2011;29(4):463-5. doi: 10.1016/j.ajem.2010.12.028 [published Online First: 2011/02/15]

For peer review only - http://bmjopen.bmj.com/site/about/guidelines.xhtml

43. Skorpen S, Nicolaisen M, Langballe EM. Hospitalisation in adults with intellectual disabilities compared with the general population in Norway. *J Intellect Disabil Res* 2016 doi: 10.1111/jir.12255 [published Online First: 2016/02/26]
44. Straetmans JM, van Schrojenstein Lantman-de Valk HM, Schellevis FG, et al. Health problems of people with intellectual disabilities: the impact for general practice. *Br J Gen Pract* 2007;57(534):64-6. [published Online First: 2007/01/25]
45. Hayden MF, Kim SH, DePaepe P. Health status, utilization patterns, and outcomes of persons with intellectual disabilities: review of the literature. *Ment Retard* 2005;43(3):175-95. doi: 10.1352/0047-6765(2005)43[175:hsupao]2.0.co;2 [published Online First: 2005/05/11]
46. Ruddick L. Health of people with intellectual disabilities: a review of factors influencing access to health care. *British journal of health psychology* 2005;10(Pt 4):559-70. doi: 10.1348/135910705x43769 [published Online First: 2005/10/22]
47. Scheepers M, Kerr M, O'Hara D, et al. Reducing Health Disparity in People with Intellectual Disabilities: A Report from Health Issues Special Interest Research Group of the International Association for the Scientific Study of Intellectual Disabilities1. *Journal of Policy and Practice in Intellectual Disabilities* 2005;2(3-4):249-55. doi: 10.1111/j.1741-1130.2005.00037.x
48. Havercamp SM, Scandlin D, Roth M. Health disparities among adults with developmental disabilities, adults with other disabilities, and adults not reporting disability in North Carolina. *Public Health Rep* 2004;119(4):418-26. doi: 10.1016/j.phr.2004.05.006 [published Online First: 2004/06/29]
49. Havercamp SM, Scott HM. National health surveillance of adults with disabilities, adults with intellectual and developmental disabilities, and adults with no disabilities. *Disability and health journal* 2014 doi: 10.1016/j.dhjo.2014.11.002 [published Online First: 2015/01/18]
50. Ali A, Scior K, Ratti V, et al. Discrimination and other barriers to accessing health care: perspectives of patients with mild and moderate intellectual disability and their carers. *PloS one* 2013;8(8):e70855. doi: 10.1371/journal.pone.0070855 [published Online First: 2013/08/21]
51. Iacono T, Bigby C, Unsworth C, et al. A systematic review of hospital experiences of people with intellectual disability. *BMC Health Serv Res* 2014;14:505. doi: 10.1186/s12913-014-0505-5 [published Online First: 2014/10/26]
52. Sowney M, Barr O. The challenges for nurses communicating with and gaining valid consent from adults with intellectual disabilities within the accident and emergency care service. *J Clin Nurs* 2007;16(9):1678-86. doi: 10.1111/j.1365-2702.2006.01642.x [published Online First: 2007/04/27]
53. Llewellyn G, Vaughan C, Emerson E. Discrimination and the health of people with intellectual disabilities. International review of research in developmental disabilities: Elsevier 2015:43-72.
54. Axmon A, Sandberg M, Ahlstrom G. Gender differences in psychiatric diagnoses in older people with intellectual disability: a register study. *BMC psychiatry* 2017;17(1):192. doi: 10.1186/s12888-017-1353-8 [published Online First: 2017/05/24]
55. Hemm C, Dagnan D, Meyer TD. Identifying training needs for mainstream healthcare professionals, to prepare them for working with individuals with intellectual disabilities: a systematic review. *Journal of applied research in intellectual disabilities : JARID* 2015;28(2):98-110. doi: 10.1111/jar.12117 [published Online First: 2014/10/01]

For peer review only - http://bmjopen.bmj.com/site/about/guidelines.xhtml

56. Heslop P, Read S, Dunwoodie Stirton F. The hospital provision of reasonable adjustments for people with learning disabilities: Findings from Freedom of Information requests. *British Journal of Learning Disabilities* 2018;46(4):258-67. doi: 10.1111/bld.12244
57. Hosking FJ, Carey IM, DeWilde S, et al. Preventable Emergency Hospital Admissions Among Adults With Intellectual Disability in England. *The Annals of Family Medicine* 2017;15(5):462-70. doi: 10.1370/afm.2104
58. Ervin DA, Hennen B, Merrick J, et al. Healthcare for Persons with Intellectual and Developmental Disability in the Community. *Frontiers in public health* 2014;2(83) doi: 10.3389/fpubh.2014.00083
59. Trollor JN, Eagleson C, Turner B, et al. Intellectual disability health content within nursing curriculum: An audit of what our future nurses are taught. *Nurse Educ Today* 2016;45:72-9. doi: 10.1016/j.nedt.2016.06.011 [published Online First: 2016/07/19]
60. Trollor JN, Ruffell B, Tracy J, et al. Intellectual disability health content within medical curriculum: an audit of what our future doctors are taught. *BMC medical education* 2016;16:105. doi: 10.1186/s12909-016-0625-1 [published Online First: 2016/04/14]
61. Sullivan WF, Berg JM, Bradley E, et al. Primary care of adults with developmental disabilities: Canadian consensus guidelines. *Can Fam Physician* 2011;57(5):541-53, e154-68. [published Online First: 2011/05/17]
62. Carey IM, Shah SM, Hosking FJ, et al. Health characteristics and consultation patterns of people with intellectual disability: a cross-sectional database study in English general practice. *Br J Gen Pract* 2016;66(645):e264-e70.
63. Durbin A, Balogh R, Lin E, et al. Emergency Department Use: Common Presenting Issues and Continuity of Care for Individuals With and Without Intellectual and Developmental Disabilities. *J Autism Dev Disord* 2018;48(10):3542-50. doi: 10.1007/s10803-018-3615-9
64. Meijer M, Carpenter S, Scholte F. European manifesto on basic standards of health care for people with intellectual disabilities. *Journal of Policy and Practice in Intellectual Disabilities* 2004;1(1):10-15.
65. Scholte FA. European Manifesto: basic standards of healthcare for people with intellectual disabilities. *Salud Publica Mex* 2008;50 Suppl 2:s273-6. [published Online First: 2008/05/28]
66. Balogh R, McMorris CA, Lunsky Y, et al. Organising healthcare services for persons with an intellectual disability. *The Cochrane database of systematic reviews* 2016;4:Cd007492. doi: 10.1002/14651858.CD007492.pub2 [published Online First: 2016/04/12]

Figure legends:

Figure legends:

Figure 1. Flowchart of the statistical analyses for somatic healthcare utilization (unplanned inpatient care, unplanned outpatient care, planned inpatient care, planned outpatient care) the following year.

For peer review only

For peer review only - http://bmjopen.bmj.com/site/about/guidelines.xhtml

617 **Figure 2 ROC-curves for multivariate models predicting four types of somatic specialist healthcare utilization among 7936 older people with intellectual disability (ID), using data on age, sex, previous healthcare utilization, and relevant diagnoses.**

For peer review only

Table 1 Relative risks (RRs) with 90% confidence intervals (CIs) for somatic healthcare utilization (unplanned/planned inpatient/outpatient) , for sex, age and healthcare utilization the year before, within a cohort of 7936 older people with intellectual disability (ID). All analyses are based on multivariate models adjusted for relevant diagnoses on ICD-10 block-level. Bold indicates p<0.1.

	Unplanned		Planned	
	Inpatient	Outpatient	Inpatient	Outpatient
	RR (90% CI)	RR (90% CI)	RR (90% CI)	RR (90% CI)
Men vs women	1.04 (0.99-1.08)	**0.93 (0.88-0.99)**	**0.92 (0.85-0.99)**	**0.92 (0.88-0.95)**
**Age (years)**				
50-59 vs <50	**1.30 (1.18-1.44)**	1.12 (0.97-1.28)	**1.28 (1.05-1.55)**	**0.92 (0.86-0.99)**
60-69 vs <50	**1.50 (1.35-1.66)**	1.03 (0.90-1.19)	**1.48 (1.21-1.80)**	**0.79 (0.73-0.85)**
70-79 vs <50	**1.75 (1.57-1.96)**	0.91 (0.77-1.07)	**1.56 (1.26-1.93)**	**0.75 (0.69-0.82)**
80-89 vs <50	**2.07 (1.81-2.37)**	0.96 (0.75-1.24)	0.97 (0.69-1.39)	**0.54 (0.45-0.64)**
90+ vs <50	**2.33 (1.60-3.41)**	1.12 (0.51-2.45)		0.32 (0.08-1.24)
**Planned inpatient previous year**				
1 vs 0	**1.14 (1.04-1.24)**	1.05 (0.95-1.16)	**1.90 (1.64-2.21)**	1.00 (0.95-1.06)
2 vs 0	1.07 (0.89-1.29)	1.02 (0.83-1.26)	**2.89 (2.22-3.75)**	1.06 (0.94-1.20)
3+ vs 0	**1.43 (1.09-1.88)**	0.99 (0.68-1.45)	**4.03 (2.84-5.70)**	**1.34 (1.11-1.61)**
**Planned outpatient previous year**				
1 vs 0	**1.25 (1.19-1.33)**	**1.10 (1.02-1.18)**	**2.07 (1.86-2.30)**	**1.89 (1.80-1.99)**
2-5 vs 0	**1.40 (1.31-1.50)**	**1.34 (1.23-1.45)**	**2.77 (2.46-3.13)**	**2.59 (2.45-2.73)**
6-10 vs 0	1.23 (0.97-1.56)	0.94 (0.72-1.22)	**2.66 (1.83-3.87)**	**2.87 (2.59-3.18)**
11+ vs 0	1.36 (0.99-1.88)	**1.53 (1.11-2.11)**	**3.80 (2.51-5.76)**	**2.45 (2.05-2.93)**
**Unplanned inpatient previous year**				
1 vs 0	**2.02 (1.89-2.16)**	**1.14 (1.06-1.23)**	**1.59 (1.42-1.78)**	**1.04 (1.00-1.09)**
2 vs 0	**2.38 (2.17-2.62)**	**1.20 (1.08-1.34)**	**1.78 (1.49-2.14)**	**1.08 (1.00-1.16)**
3+ vs 0	**2.70 (2.39-3.04)**	**1.19 (1.05-1.35)**	**2.43 (2.03-2.90)**	**1.16 (1.07-1.26)**
**Unplanned outpatient previous year**				
1 vs 0	**1.25 (1.17-1.33)**	**1.78 (1.65-1.91)**	**1.20 (1.06-1.35)**	1.02 (0.97-1.07)
2-5 vs 0	**1.42 (1.29-1.56)**	**2.73 (2.47-3.02)**	**1.21 (1.00-1.46)**	**1.15 (1.08-1.23)**
6-10 vs 0	**1.33 (1.01-1.74)**	**3.66 (3.05-4.39)**	1.18 (0.69-2.00)	0.97 (0.78-1.20)
11+ vs 0	**1.52 (1.09-2.13)**	**3.74 (2.90-4.82)**	**2.22 (1.48-3.34)**	**1.45 (1.11-1.89)**

For peer review only

For peer review only - http://bmjopen.bmj.com/site/about/guidelines.xhtml

Table 2 Relative risks for somatic healthcare utilization (unplanned/planned inpatient/outpatient) for people with diagnosis (A20-A29, A30-A49, etc) compared to those without, within a cohort of 7936 older people with intellectual disability (ID). All analyses are based on multivariate models adjusted for sex, age, and healthcare utilization previous year. Only results with p<0.1 are included.

	Unplanned		Planned	
	In-patient	Out-patient	In-patient	Out-patient
A20-A28: Certain zoonotic bacterial diseases				2.25
A30-A49: Other bacterial diseases				
A50-A64: Infections with a predominantly sexual mode of transmission	10.78			2.83
A90-A99: Arthropod-borne viral fevers and viral haemorrhagic fevers				1.45
B20-B24: Human immunodeficiency virus [HIV] disease	2.21			
B25-B34: Other viral diseases				
B50-B64: Protozoal diseases	4.29			
B95-B98: Bacterial, viral and other infectious agents		0.68		
B99-B99: Other infectious diseases				0.69
C00-C97: Malignant neoplasms	0.77			1.43
D00-D09: In situ neoplasms	2.18			
D80-D89: Certain disorders involving the immune mechanism				1.72
E10-E14: Diabetes mellitus	1.27	1.18		1.39
E15-E16: Other disorders of glucose regulation and pancreatic internal secretion		2.59		
E70-E90: Metabolic disorders	0.79	0.73		
F00-F09: Organic, including symptomatic, mental disorders				0.79
F10-F19: Mental and behavioural disorders due to psychoactive substance use	1.56	1.55		
F40-F48: Neurotic, stress-related and somatoform disorders		1.28		
F50-F59: Behavioural syndromes associated with physiological disturbances and physical factors	1.78			
F70-F79: Mental retardation		0.91		0.82
F80-F89: Disorders of psychological development		0.91		0.81
F99-F99: Unspecified mental disorder				
G20-G26: Extrapyramidal and movement disorders	1.32	1.38		1.36
G40-G47: Episodic and paroxysmal disorders	1.62	1.53		1.49
G60-G64: Polyneuropathies and other disorders of the peripheral nervous system	1.84			
G70-G73: Diseases of myoneural junction and muscle		1.93		
G80-G83: Cerebral palsy and other paralytic syndromes	1.31			
H15-H22: Disorders of sclera, cornea, iris and ciliary body		1.37		
H25-H28: Disorders of lens		0.81	1.37	
H30-H36: Disorders of choroid and retina				1.19
H40-H42: Glaucoma				1.61

For peer review only

For peer review only - http://bmjopen.bmj.com/site/about/guidelines.xhtml

H65-H75: Diseases of middle ear and mastoid				1.62
H90-H95: Other disorders of ear		1.37		
I10-I15: Hypertensive diseases				
I20-I25: Ischaemic heart diseases	1.17			1.12
I30-I52: Other forms of heart disease				1.17
I60-I69: Cerebrovascular diseases				
I80-I89: Diseases of veins, lymphatic vessels and lymph nodes, not elsewhere classified				0.83
J00-J06: Acute upper respiratory infections	1.28			0.77
J09-J18: Influenza and pneumonia				0.84
J20-J22: Other acute lower respiratory infections				
J30-J39: Other diseases of upper respiratory tract				1.28
J40-J47: Chronic lower respiratory diseases	1.19			
J60-J70: Lung diseases due to external agents				0.78
J95-J99: Other diseases of the respiratory system				
K40-K46: Hernia				
K50-K52: Noninfective enteritis and colitis		1.42		1.44
K55-K63: Other diseases of intestines	1.22	1.17	1.37	
K65-K67: Diseases of peritoneum				
K70-K77: Diseases of liver	1.35			1.46
L10-L14: Bullous disorders		2.70		
L20-L30: Dermatitis and eczema	1.46	1.66		
L50-L54: Urticaria and erythema	1.98			
L80-L99: Other disorders of the skin and subcutaneous tissue	0.64			
M00-M25: Arthropathies	1.13			1.22
M95-M99: Other disorders of the musculoskeletal system and connective tissue		2.39		
N00-N08: Glomerular diseases				1.51
N10-N16: Renal tubulo-interstitial diseases				0.72
N17-N19: Renal failure				1.16
N30-N39: Other diseases of urinary system	1.04			
N40-N51: Diseases of male genital organs	0.54			
P20-P29: Respiratory and cardiovascular disorders specific to the perinatal period	0.63			
Q20-Q28: Congenital malformations of the circulatory system	1.59			
Q60-Q64: Congenital malformations of the urinary system				1.50
Q90-Q99: Chromosomal abnormalities, not elsewhere classified		0.84		
R00-R09: Symptoms and signs involving the circulatory and respiratory systems	1.29	1.50		
R10-R19: Symptoms and signs involving the digestive system and abdomen	1.22	1.22		
R50-R69: General symptoms and signs				
S00-S09: Injuries to the head	1.16			
S30-S39: Injuries to the abdomen, lower back, lumbar spine and pelvis				
S60-S69: Injuries to the wrist and hand		1.18		
S80-S89: Injuries to the knee and lower leg		0.84		
T15-T19: Effects of foreign body entering through natural orifice	1.31			
T51-T65: Toxic effects of substances chiefly nonmedicinal as to source	2.00			

For peer review only

Z70-Z76: Persons encountering health services in other circumstances		1.29		

For peer review only

For peer review only - http://bmjopen.bmj.com/site/about/guidelines.xhtml

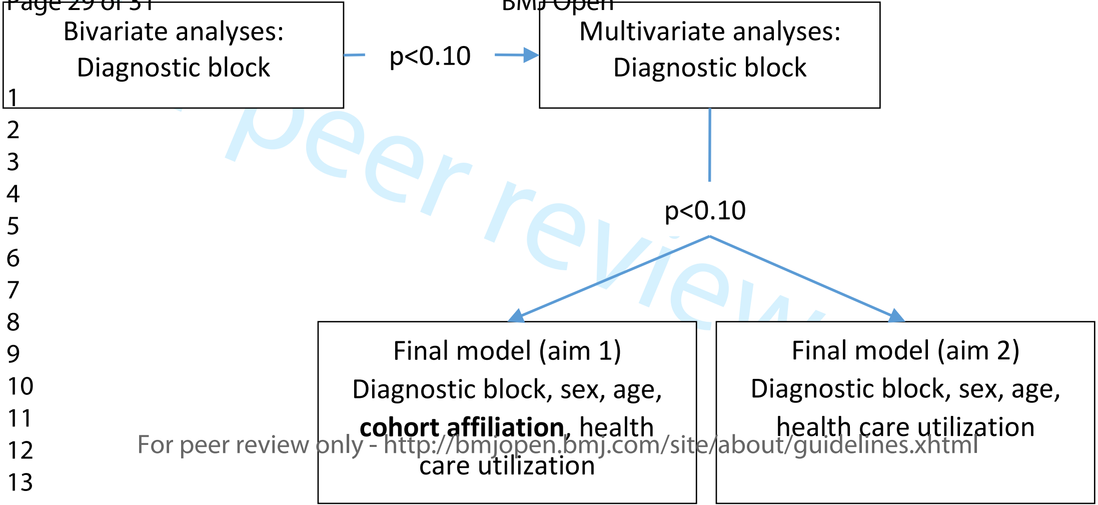

For peer review only - http://bmjopen.bmj.com/site/about/guidelines.xhtml

Unplanned inpatient care

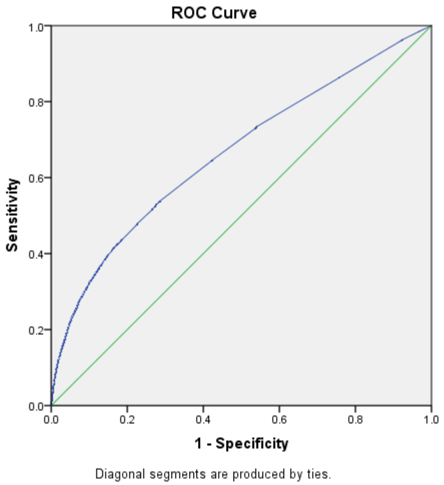

Unplanned outpatient care

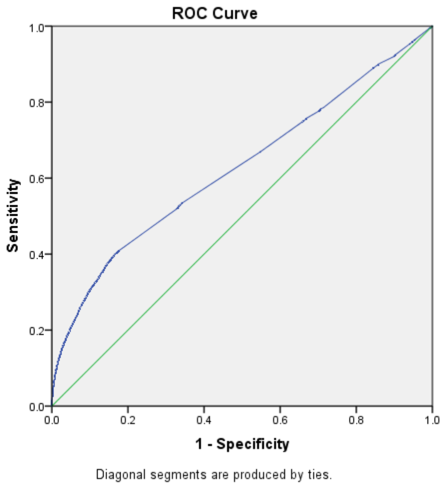

Planned inpatient care

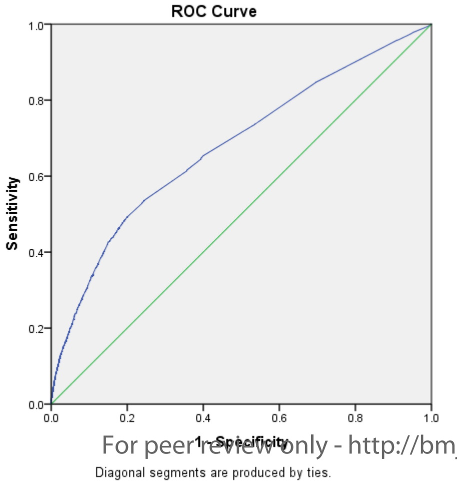

Planned outpatient care

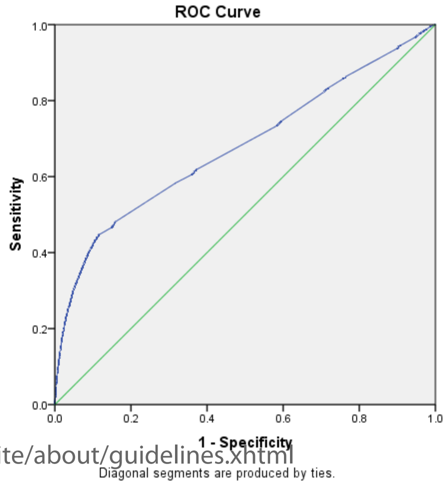

#1STROBE 2007 (v4) Statement—Checklist of items that should be included in reports of *cohort studies*

Section/Topic	Item #	Recommendation	Reported on page #
**Title and abstract**	1	(*a*) Indicate the study's design with a commonly used term in the title or the abstract	#1/Title page
		(*b*) Provide in the abstract an informative and balanced summary of what was done and what was found	#2-3
**Introduction**			
Background/rationale	2	Explain the scientific background and rationale for the investigation being reported	#6-7
Objectives	3	State specific objectives, including any prespecified hypotheses	#7
**Methods**			
Study design	4	Present key elements of study design early in the paper	#2 & #8
Setting	5	Describe the setting, locations, and relevant dates, including periods of recruitment, exposure, follow-up, and data collection	#8-9
Participants	6	(*a*) Give the eligibility criteria, and the sources and methods of selection of participants. Describe methods of follow-up	#8-9
		(*b*) For matched studies, give matching criteria and number of exposed and unexposed	#8-9
Variables	7	Clearly define all outcomes, exposures, predictors, potential confounders, and effect modifiers. Give diagnostic criteria, if applicable	#8-11
Data sources/ measurement	8*	For each variable of interest, give sources of data and details of methods of assessment (measurement). Describe comparability of assessment methods if there is more than one group	#9
Bias	9	Describe any efforts to address potential sources of bias	#9-11
Study size	10	Explain how the study size was arrived at	#8-9
Quantitative variables	11	Explain how quantitative variables were handled in the analyses. If applicable, describe which groupings were chosen and why	#9-11
Statistical methods	12	(*a*) Describe all statistical methods, including those used to control for confounding	#9-11
		(*b*) Describe any methods used to examine subgroups and interactions	#9
		(*c*) Explain how missing data were addressed	N/A
		(*d*) If applicable, explain how loss to follow-up was addressed	N/A
		(*e*) Describe any sensitivity analyses	N/A
**Results**			

For peer review only

Participants	13*	(a) Report numbers of individuals at each stage of study—eg numbers potentially eligible, examined for eligibility, confirmed eligible, included in the study, completing follow-up, and analysed	#9
		(b) Give reasons for non-participation at each stage	N/A
		(c) Consider use of a flow diagram	N/A
Descriptive data	14*	(a) Give characteristics of study participants (eg demographic, clinical, social) and information on exposures and potential confounders	N/A
		(b) Indicate number of participants with missing data for each variable of interest	N/A
		(c) Summarise follow-up time (eg, average and total amount)	N/A
Outcome data	15*	Report numbers of outcome events or summary measures over time	N/A
Main results	16	(*a*) Give unadjusted estimates and, if applicable, confounder-adjusted estimates and their precision (eg, 95% confidence interval). Make clear which confounders were adjusted for and why they were included	#11, Table 1, Table 2, Figure 2
		(*b*) Report category boundaries when continuous variables were categorized	#10
		(*c*) If relevant, consider translating estimates of relative risk into absolute risk for a meaningful time period	N/A
Other analyses	17	Report other analyses done—eg analyses of subgroups and interactions, and sensitivity analyses	N/A
**Discussion**			
Key results	18	Summarise key results with reference to study objectives	#13-15
**Limitations**			
Interpretation	20	Give a cautious overall interpretation of results considering objectives, limitations, multiplicity of analyses, results from similar studies, and other relevant evidence	#12-13
Generalisability	21	Discuss the generalisability (external validity) of the study results	
**Other information**			
Funding	22	Give the source of funding and the role of the funders for the present study and, if applicable, for the original study on which the present article is based	#3

*Give information separately for cases and controls in case-control studies and, if applicable, for exposed and unexposed groups in cohort and cross-sectional studies.

Note: An Explanation and Elaboration article discusses each checklist item and gives methodological background and published examples of transparent reporting. The STROBE checklist is best used in conjunction with this article (freely available on the Web sites of PLoS Medicine at http://www.plosmedicine.org/, Annals of Internal Medicine at http://www.annals.org/, and Epidemiology at http://www.epidem.com/). Information on the STROBE Initiative is available at www.strobe-statement.org.

For peer review only
